# Phytosterol-induced modulation of gut microbial bile salt hydrolases ameliorates hyperlipidemia via taurohyodeoxycholic acid-mediated FXR antagonism

**DOI:** 10.1080/19490976.2026.2731687

**Published:** 2026-09-17

**Authors:** Jiayue Xia, Da Pan, Tianyu Wu, Zhiyuan Feng, Yanyan Tian, Zhi He, Zhenzhen Zhang, Yingqi Shao, Junhui Yu, Wei Yan, Jiongnan Wang, Shiyu Yin, Xingyi Jin, Yilu Chen, Yuanyuan Wang, Guiju Sun

**Affiliations:** a Key Laboratory of Environmental Medicine and Engineering of Ministry of Education, School of Public Health, Southeast University, Nanjing, People's Republic of China; b Department of Nutrition and Food Hygiene, School of Public Health, Southeast University, Nanjing, People's Republic of China; c Zhongda Hospital Affiliated to Southeast University, Nanjing, People's Republic of China

**Keywords:** Phytosterols, bile salt hydrolase, gut microbiota, taurohyodeoxycholic acid, FXR antagonism, dyslipidemia

## Abstract

Dyslipidemia remains a major cardiovascular risk factor. Although dietary phytosterols have established lipid-lowering effects, their interactions with the gut-liver metabolic axis remain incompletely understood. Here, we integrated human observational and intervention studies with mechanistic experiments in hyperlipidemic rats, fecal microbiota transplantation (FMT), and in vitro cellular models to investigate how phytosterols influence lipid homeostasis through gut microbiota-bile acid signaling. Human analyses identified associations between phytosterol exposure, lipid phenotypes, gut microbial features, and circulating bile acid profiles. In rats, phytosterol treatment altered gut microbial composition and the relative abundance of putative bile salt hydrolase (BSH)-producing taxa, accompanied by reduced ileal luminal BSH activity. These changes coincided with bile acid remodeling, including increased concentrations of taurohyodeoxycholic acid (THDCA) in liver tissue and ileal contents. In vitro cellular assays indicated that THDCA antagonized intestinal farnesoid X receptor (FXR) signaling. Consistent with this, phytosterol treatment in vivo attenuated ileal FXR-fibroblast growth factor 15 (FGF15) signaling and decreased hepatic CYP7A1 while increasing CYP7B1 protein expression, a pattern consistent with a shift from the classical toward the alternative bile acid synthesis pathway. Complementary FMT experiments further supported a contributory role of the gut microbiota in the lipid-modulating effects of phytosterols. Collectively, these findings suggest that dietary phytosterols may ameliorate dyslipidemia partly through modulation of the gut microbiota-bile acid-FXR axis, with microbiota-associated BSH-THDCA-FXR signaling representing one plausible contributing pathway. This study provides convergent preclinical mechanistic and exploratory human evidence for a diet-microbe-host pathway relevant to dyslipidemia.

## Introduction

Dyslipidemia is a highly prevalent metabolic disorder and remains one of the leading modifiable risk factors for cardiovascular disease (CVD) worldwide.^1^ ^,^ ^2^ With the ongoing aging of the population and the widespread adoption of Westernized dietary patterns, the burden of dyslipidemia continues to rise, imposing substantial health and socioeconomic costs.^3^ ^,^ ^4^ Although pharmacological therapies, particularly statins, represent the cornerstone of lipid-lowering treatment,^5^ their long-term use is frequently constrained by adverse effects, interindividual variability in therapeutic response, issues of adherence, and limited accessibility in certain populations,^6^ which underscores the need for safe, accessible strategies. Consequently, lifestyle modifications, especially dietary intervention, remain the first line and most universally applicable strategy for the prevention and management of dyslipidemia.^7^

Among dietary bioactive compounds, phytosterols (PS), the plant-derived sterols abundant in vegetable oils, nuts, and seeds, have garnered significant attention due to their structural similarity to cholesterol.^8^ Because PS cannot be synthesized endogenously, humans must obtain them entirely from the diet. It is classically understood that PS lower serum cholesterol by competitively inhibiting intestinal cholesterol absorption through displacement of cholesterol from mixed micelles.^9^ This mechanism has formed the basis for the development of PS-fortified foods and dietary supplements, which are widely recommended as adjuncts to lipid-lowering therapy. However, accumulating evidence indicates that the lipid-lowering efficacy of PS exhibits marked interindividual variability, and that substantial metabolic benefits can be observed despite their extremely low systemic bioavailability.^10^ These observations suggest that mechanisms beyond simple luminal competition for cholesterol absorption may contribute to their physiological effects. In our previous randomized controlled trial (RCT), we demonstrated that a high-PS diet (delivered via enriched cooking oil) significantly reduced serum total cholesterol (TC), triglycerides (TG), and low-density lipoprotein cholesterol (LDL-C), thereby lowering the estimated 10-year CVD risk in Chinese adults with dyslipidemia.^11^ Importantly, this intervention reflected real-world dietary patterns rather than isolated nutrient supplementation, underscoring the translational relevance of PS as a feasible nutritional strategy. Despite this confirmed clinical efficacy, the precise molecular and metabolic pathways mediating these lipid-lowering effects remain incompletely understood, particularly with respect to host-microbiome interactions and enterohepatic regulation.^12^

Emerging evidence implicates the gut microbiota has emerged as a pivotal regulator of host lipid metabolism, largely through the modulation of the bile acid (BA) pool and enterohepatic circulation.^13^ Our recent integrative multi-omics analyzes revealed that dyslipidemia is intrinsically associated with substantial alterations in gut microbial community structure and BA metabolic profiles.^14^ Gut bacteria govern the conversion of primary BAs into secondary BAs via enzymatic processes such as bile salt hydrolase (BSH)-mediated deconjugation, thereby shaping the pool of bioactive BA species.^15^ These BAs function not only as detergents facilitating lipid absorption but also as potent signaling molecules that activate nuclear receptors, most notably the farnesoid X receptor (FXR).^16^ In the ileum, FXR activation induces fibroblast growth factor 15/19 (FGF15 in rodents, FGF19 in humans), which acts in an endocrine manner to suppress hepatic cholesterol 7α-hydroxylase (CYP7A1), the rate-limiting enzyme in BA synthesis, thereby tightly linking BA signaling to cholesterol homeostasis.^17^ Accordingly, the gut microbiota-BA-FXR axis has been increasingly recognized as a promising therapeutic target for the management of hypercholesterolemia and metabolic diseases.^18^

Despite these advances, whether dietary PS exerts their lipid-lowering effects through remodeling of the gut microbiota and subsequent alterations in BA composition and FXR signaling remains largely unexplored. It is unknown whether PS, when consumed as part of a realistic dietary matrix, can selectively modulate microbial functions relevant to BA metabolism and thereby engage enterohepatic signaling pathways that contribute to systemic lipid regulation. Here, by integrating population-based epidemiological analysis with a pragmatic dietary intervention, we systematically investigated the lipid-lowering efficacy and microbial-metabolic impact of dietary PS in a real-world context. Subsequent mechanistic dissection in animal models and validation through a RCT elucidated a gut microbiota-BA-FXR axis driving these benefits. This study bridges molecular mechanisms with clinical applicability and provides a compelling scientific rationale for the development and implementation of PS-fortified foods as a practical and scalable nutritional strategy for dyslipidemia management.

## Material and methods

### Antibodies and critical commercial assays

Primary antibodies used in this study were as follows: CYP7A1 rabbit polyclonal antibody (Cat#A22897; RRID: AB_3105781; ABclonal), CYP7B1 rabbit polyclonal antibody (Cat#A17872; RRID: AB_2769104; ABclonal), FXR/NR1H4 rabbit monoclonal antibody (Cat#A24015; RRID:N/A; ABclonal), FGF19 rabbit polyclonal antibody (Cat#A6589; RRID:AB_2767182; ABclonal), SHP/NR0B2 rabbit polyclonal antibody (Cat#A16454; RRID:AB_2770666; ABclonal), anti‑β‑actin rabbit polyclonal antibody (Cat#GB11001-100; RRID:N/A; Servicebio), and goat anti-rabbit IgG (H+L) secondary antibody (Cat#G1213; RRID:N/A; Servicebio). Commercially available assay kits were listed as follows: serum lipid ELISA kits for rats (Nanjing Jiancheng Bioengineering Institute); ELISA kits targeting rat C-reactive protein, interleukin-1β, interleukin-6 and tumor necrosis factor-*α* (Nanjing Lapuda Biotechnology Co., Ltd.).

## Ethics statement

All experiments involving animals were conducted according to the ethical policies and procedures approved by the Animal Research Ethics Review Committee of Southeast University (Approval no. 20231017003). All human participant-related studies in this work were approved by the Ethics Committee of Zhongda Hospital Affiliated to Southeast University (Approval No. 2024ZDSYLL478-P01) and the Ethics Committee of Dongtai People’s Hospital (Approval No. 2023-dtry-K048-PJ), respectively.

### Human studies

#### Study participants

The present human studies encompass one human observational study and two independent PS intervention clinical trials. Human observational study primarily investigated the potential association between dietary PS intake and blood lipid levels. Trial 1 recruited a cohort of patients diagnosed with dyslipidemia. Trial 2 targeted subjects with borderline hypercholesterolemia, enrolling individuals who met the diagnostic criteria for borderline-high serum cholesterol for analysis. Both trials were conducted in strict adherence to the ethical principles outlined in the Declaration of Helsinki. The study protocols and informed consent documentation were reviewed and approved by the respective local research ethics committees. Written informed consent was obtained from all participants prior to the commencement of screening procedures.

For human observational study, this study recruited in-service employees from Nanjing, Jiangsu Province, China, between March and December 2023. Eligible participants were permanent residents aged 18–65 y who provided written informed consent. Individuals were excluded if they were non-permanent residents, had limited physical mobility, or demonstrated communication barriers precluding study compliance. For the final analysis, participants were further excluded based on the following quality control criteria: (1) incomplete questionnaires or missing dietary data; (2) implausible total energy intake (defined as <800 or >6,000 kcal/day for men and <600 or >4,000 kcal/day for women); or (3) non-fasting blood samples. A total of 183 subjects were enrolled and categorized into two groups based on their lipid profiles: a healthy control group (*n* = 98) and a dyslipidemia group (*n* = 85). PS intake was classified into three levels based on quartiles: low (<*P*_25_, reference), moderate (*P*_25_-*P*_75_), and high (>*P*_75_).^19^ This observational study was approved by the Ethics Committee of Zhongda Hospital Affiliated to Southeast University (Approval No. 2024ZDSYLL478-P01) and was conducted in accordance with the Declaration of Helsinki.

For clinical trial 1, a randomized, single-blind, controlled feeding trial (Registration: ChiCTR2200059288) was conducted in Nanjing, Jiangsu, China, between April 2023 and September 2023. 104 patients with dyslipidemia were randomized (1:1) to receive a high-PS diet (HPS) intervention or a low-PS diet (LPS) control group at the employee cafeteria of a corporate entity for three months after following a two-week run-in period. The specific details of the clinical trial are as described in our previous study.^11^ The vegetable oils given are a major source of PS in the Chinese diet, we achieved HPS and LPS diets by substituting daily cooking oils, while providing identical menus to both groups. The HPS group replaced daily cooking oil with high-PS blended oil composed of corn germ oil and wheat germ oil (14,320 mg/kg PS), whereas the LPS group utilized low-PS peanut oil (3,153 mg/kg PS). Lunches were centrally prepared and standardized to ensure isocaloric intake, while the oil consumption for other meals was strictly controlled and monitored using the two types of cooking oils provided to both groups (which had no visible differences), with a target of 2,000 kcal per day. Based on the prescribed 2000-kcal daily dietary target, the theoretical phytosterol intake contributed solely by cooking oil per lunch was estimated at 69.36 mg in the LPS group and 315.04 mg in the HPS group. Primary outcomes comprised changes in serum lipid parameters: TC, TG, LDL-C, high-density lipoprotein cholesterol (HDL-C), and non-HDL-C. During the intervention, a total of 5 participants discontinued the intervention. All participants were included in the intention-to-treat (ITT) analysis. The per-protocol (PP) population consisted of subjects who adhered to the trial protocol.

For clinical trial 2, Serum samples were obtained from a previously conducted randomized, triple-blind, placebo-controlled trial (Registration: ChiCTR2400085771) at Dongtai People’s Hospital (Jiangsu, China).^20^ The present study represents a secondary analysis of banked serum samples collected during the same previously approved and registered clinical trial^20^; therefore, the ethical approval number for this component of the present study is the same as that reported in the previous publication. No additional participants were recruited, and no new randomization or clinical intervention was conducted for the present analysis. Detailed methodologies regarding participant recruitment, randomization, intervention procedures, and the primary clinical outcomes have been reported previously.^20^ Briefly, the original trial recruited 88 participants with borderline hypercholesterolemia. For the present analysis, we focused specifically on the independent effects of PS and included serum samples from the PS group (2 g PS/day) and the placebo group. Samples collected at baseline and the 1 month of intervention were analyzed to characterize phytosterol-associated changes in serum BA profiles.

#### Dietary assessment and estimated phytosterol intake

Demographic information, including name, sex, age, ethnicity, and education level, history of chronic diseases, family history, and self-reported smoking and alcohol consumption status, were collected through face-to-face interviews. Physical activity was assessed using the long form of the International Physical Activity Questionnaire (IPAQ), and levels were expressed as metabolic equivalent min per week (MET-min/week). Dietary history was assessed using a semi-quantitative Food Frequency Questionnaire (FFQ), which comprises 14 major food categories. These categories included: (1) rice and rice products (e.g., steamed rice, rice noodles); (2) wheat flour and its products (e.g., steamed buns, noodles); (3) tubers and roots (e.g., potatoes, taro, sweet potatoes); (4) traditional fried wheat-based foods (e.g., fried cakes, sesame balls, deep-fried dough sticks, pancakes, twisted dough); (5) coarse grains (e.g., millet, corn, sorghum, mung beans, red beans); (6) fruits; (7) edible fungi/mushrooms (e.g., enoki mushrooms, wood ear, shiitake, oyster mushrooms); (8) vegetables (dark green leafy vegetables, orange/red vegetables, and others); (9) meat and poultry (red meat, poultry, organ meats, eggs, and aquatic products); (10) legumes and soy products (e.g., soybeans, tofu, dried tofu, tofu pudding, soy milk, dried bean curd sticks, bean curd sheets); (11) nuts and seeds (e.g., sunflower seeds, peanuts, walnuts, pistachios, hazelnuts); (12) dairy and dairy products; (13) vegetable oils; and (14) animal fats. Participants reported their dietary habits over the past year by recalling the frequency of consumption (daily, weekly, monthly, or yearly) and the average portion size for each food item. Based on the daily average intake of each food item and nutrient data from the 6th Edition of the China Food Composition Tables (Table S1), the daily intakes of PS were calculated, including *β*-sitosterol (mg), campesterol (mg), stigmasterol (mg), *β*-sitostanol (mg), and campestanol (mg). Total PS intake was defined as the sum of these individual PS species. The specific PS-containing food items included in the questionnaire are listed in Table S2.

#### Diagnostic criteria for dyslipidemia and borderline high blood cholesterol

All participants provided overnight fasting blood samples (≥12 h fasting) for serum lipid quantification to support dyslipidemia screening and diagnosis. Distinct diagnostic thresholds were adopted for the two human trials to meet their separate enrollment objectives. Trial 1 recruited patients with clinically confirmed dyslipidemia while Trial 2 enrolled subjects with mild borderline hypercholesterolemia. In the human observational study and Clinical Trial 1, dyslipidemia was diagnosed according to the 2016 Chinese Guideline for the Management of Dyslipidemia in Adults.^21^ Dyslipidemia was diagnosed if any of the following criteria were met: serum TC ≥ 5.2 mmol/L, TG ≥ 1.7 mmol/L, HDL-C < 1.0 mmol/L, or LDL-C ≥ 3.4 mmol/L. In Clinical Trial 2, participants were diagnosed with borderline hypercholesterolemia if they simultaneously met the criteria for TC, TG, and non-HDL-C, or for LDL-C, TG, and non-HDL-C. The specific cut-off ranges were as follows: 5.2 mmol/L ≤ serum TC < 6.2 mmol/L, 3.4 mmol/L ≤ serum LDL-C < 4.1 mmol/L, 1.7 mmol/L ≤ serum TG < 2.3 mmol/L, and 4.1 mmol/L ≤ serum non-HDL-C < 4.9 mmol/L.

#### Phytosterol preparation

To explore the mechanistic effects of dietary PS in animal models, high-purity PS powder was extracted from the wheat and corn germ blended oil applied in the clinical trial for subsequent animal interventions. In brief, the deodorizer distillate produced during physical deodorization of crude oil was subjected to methyl esterification. Following phase separation, the aqueous fraction was precipitated via calcium soap formation, while the upper oily fraction was refluxed with sodium methoxide and methanol at 343 K for 120 min. The mixture was subsequently neutralized with hydrochloric acid, rinsed with hot water, and re-separated. The obtained methyl-esterified distillate was cooled to 277 K for crystallization and centrifugation. The precipitated solid fraction was recrystallized, and the residual oily fraction was concentrated to obtain refined PS powder with a final purity of 96.1%. All PS extraction and purification procedures were performed by Wilmar Biotechnology Co., Ltd (Jiangsu, China).

### Animal experiments

To investigate the effects and potential mechanisms of PS on lipid metabolism, five independent animal experiments were conducted sequentially: dose-response validation, therapeutic intervention, fecal microbiota transplantation (FMT), pharmacological intervention targeting FXR signaling, and individual BA supplementation.

#### Sample size

Sample sizes for the animal experiments were determined based on previous studies using comparable high-fat diet-induced dyslipidemia models and related intervention paradigms, together with experimental feasibility and animal-welfare considerations. No formal a priori power calculation was performed. The planned group sizes for each experiment are specified in the corresponding experimental descriptions below.

No formal a priori inclusion or exclusion criteria were established. Animals that died before the scheduled study endpoint were excluded from terminal biochemical and histological analyzes but were retained in survival analyzes where applicable. No individual data points were excluded solely on the basis of the magnitude or direction of the observed outcomes.

#### Randomization

Animals were randomly allocated to experimental groups using a computer-generated randomization sequence before the start of each intervention.

#### Anesthesia and euthanasia

For terminal sample collection in all animal experiments, rats were anesthetized with isoflurane delivered in oxygen. Anesthesia was induced with 3%–4% isoflurane in an induction chamber equipped with a calibrated precision vaporizer and waste anesthetic gas scavenging system. Following loss of the pedal withdrawal reflex, anesthesia was maintained with 1.5%–2.0% isoflurane delivered via a nose cone. Anesthetic depth was monitored by periodic assessment of the pedal withdrawal reflex and respiratory rate. Once a surgical plane of anesthesia was confirmed, terminal blood samples were collected from the abdominal aorta while the animals remained under continuous isoflurane anesthesia. Rats were subsequently euthanized by exsanguination while maintained under deep isoflurane anesthesia. Death was confirmed by the permanent cessation of spontaneous respiration and heartbeat, observed continuously for at least 60 s. Tissue collection was performed only after death had been confirmed. All anesthesia and euthanasia procedures were conducted in accordance with the approved institutional animal experimental protocol and applicable animal welfare and veterinary guidelines.

#### Rats model and pseudo-germ-free rat preparation

All rats were housed in a specific pathogen-free (SPF) facility under a 12-h light/dark cycle with ad libitum access to food and water. 7-week-old male Sprague-Dawley (SD) rats were purchased from Jinan Pengyue Experimental Animal Breeding Co., Ltd. (Shandong, China; Production License: SCXK (Lu) 2022-0006). To induce hyperlipidemia, rats in the model group were fed a 40% high-fat diet (HFD) for 14 weeks. The HFD was manufactured by Jiangsu Xietong Pharmaceutical Bio-engineering Co., Ltd. (Feed Production License: Su Si Zheng [2024] 01008). Detailed compositions of the control and high-fat model diets are provided in Table S3.

To establish pseudo‑germ-free recipients, rats received daily intragastric gavage of a mixed antibiotic cocktail for two consecutive weeks. The cocktail consisted of metronidazole (1g/L), vancomycin hydrochloride (0.5g/L), neomycin sulfate (1g/L), and ampicillin (1g/L). The solution was freshly prepared daily and stored in the dark. Rats administered the antibiotic solution via oral gavage at a dose of 0.5 mL/kg every night for two consecutive weeks to deplete gut microbiota. To validate the establishment of the pseudo-germ-free rat model, fecal samples were collected at baseline and after two weeks of treatment with the quadruple antibiotic cocktail. These samples were snap-frozen in liquid nitrogen and stored at −80 °C. Absolute quantitative qPCR was employed for assessment. Specifically, the total bacterial load was determined through absolute qPCR, with pseudo-GF rat model success defined as a post-treatment bacterial load of <10% of baseline levels, corresponding to a depletion efficiency of >90%.

#### Animal experiment 1: Gradient-dose of phytosterol intervention

A total of 50 male Sprague-Dawley (SD) rats aged 7 weeks were initially randomly allocated to five groups (*n* = 10 per group): normal control diet (NCD), HFD, and three HFD+PS groups. Rats in the NCD group received 0.5% sodium carboxymethyl cellulose (CMC-Na) by oral gavage, whereas rats in the HFD group were gavaged with 0.5% CMC-Na. Rats in the HFD+PS groups were fed a HFD and orally administered PS suspended in 0.5% CMC-Na at doses of 200, 500, or 1,000 mg/kg/day for 13 weeks. At the end of the intervention, six animals per group were included in subsequent analyzes. No additional animals or data points were excluded. Terminal blood and tissue collection and euthanasia were performed as described above, and liver and intestinal tissues were collected for histological assessment.

#### Animal experiment 2: Therapeutic phytosterol treatment

After 14 weeks of HFD feeding to induce dyslipidemia, 24 male SD rats aged 7 weeks were randomly assigned to three groups (*n* = 8 per group) for a subsequent 13-week intervention: NCD group, in which rats were switched to a standard control diet and administered 0.5% CMC-Na vehicle; HFD group, in which rats remained on the HFD and received 0.5% CMC-Na vehicle; and HFD+PS group, in which rats remained on the HFD and were orally administered PS suspended in 0.5% CMC-Na at 500 mg/kg/day. All treatments were administered once daily by oral gavage. At the end of the intervention, terminal blood and tissue collection and euthanasia were performed as described above. Liver and ileal tissues were collected for Western blotting, targeted metabolomics, and histological analyzes, and ileal contents were collected for gut microbiota profiling.

#### Animal experiment 3: FMT experiment

Microbiota donors were selected from animal experiment 2. After 8 weeks of intervention, fresh fecal samples were collected from rats in the HFD and HFD+PS groups. The feces were suspended in sterile phosphate-buffered saline (PBS), homogenized, and filtered through sterile gauze to obtain fecal suspensions for transplantation. Recipient animals were 21-week-old male hyperlipidemic SD rats (*n* = 14), which were housed individually in group-separated sterile isolators and maintained on an autoclaved HFD throughout the experiment. To deplete the endogenous gut microbiota and establish an antibiotic-treated pseudo-germ-free model, recipient rats were orally gavaged once daily with a quadruple-antibiotic cocktail containing metronidazole, vancomycin hydrochloride, neomycin sulfate, and ampicillin at concentrations of 1, 0.5, 1, and 1 g/L, respectively, for 2 weeks. After antibiotic treatment, recipient rats underwent a 3-day antibiotic-free washout period and were then randomly assigned to two groups (*n* = 7 per group): the HFD-FMT group, which received fecal microbiota from HFD-fed donors, and the HFD+PS-FMT group, which received fecal microbiota from HFD+PS-treated donors. Recipient rats were gavaged daily with 2 mL of freshly prepared fecal suspension for 14 consecutive days. After FMT, all rats were maintained on the autoclaved HFD for an additional 2 weeks. At the end of the experiment, terminal blood and tissue collection and euthanasia were performed as described above. Liver and intestinal tissues were collected for Western blotting and histological assessment.

#### Animal experiment 4: FXR pharmacological intervention

For the FXR pharmacological intervention experiment, 21-week-old male SD rats were randomly assigned to seven groups (*n* = 8 per group): NCD, HFD, HFD+PS, HFD+guggulsterone(GS), HFD+GS+PS, HFD+ursodeoxycholic acid (UDCA), and HFD+UDCA+PS. Rats in the PS-treated groups were orally administered PS at 500 mg/kg/day, and rats in the GS- or UDCA-treated groups received GS or UDCA at 50 mg/kg/day, respectively. After 8 weeks of intervention, terminal blood and tissue collection and euthanasia were performed as described above, and liver and ileal tissues were harvested for downstream analyzes.

#### Animal experiment 5: HDCA/THDCA supplementation

For the bile acid supplementation experiment, 21-week-old male SD rats were randomly allocated to four groups (*n* = 7 per group): NCD, HFD, HFD+hyodeoxycholic acid (HDCA), and HFD+taurohyodeoxycholic acid (THDCA). Rats in the HDCA and THDCA groups were orally gavaged with HDCA or THDCA at 75 mg/kg/day for 5 weeks. This dose was chosen based on commonly used dosing ranges in published rodent BA intervention studies. At the end of the 5-week intervention, terminal blood and tissue collection and euthanasia were performed as described above, and liver and ileal tissues were harvested for Western blotting and histological assessment.

#### Preparation of fecal microbiota suspensions

Fresh fecal samples (200–350 mg) were collected from donor rats of the HFD and PS groups in animal experiment 2 and transferred into 50 mL cryotubes. Fresh feces collected from all donors within the same group were pooled to prepare a single batch of microbial suspension. All subsequent processing steps were performed within an anaerobic workstation to maintain viability of obligate and facultative anaerobic BSH-producing microbes. Sterile PBS containing 0.5 g/L L-cysteine and 0.2 g/L sodium sulfide (reducing agents) was pre-equilibrated anaerobically overnight to remove dissolved oxygen prior to use. Fecal material was resuspended in the pre-reduced PBS at a final ratio of ~200 mg feces per 2 mL buffer, followed by thorough homogenization with steel beads in a tissue grinder. The homogenate was centrifuged at 2500 rpm for 1 min at 4°C to remove coarse particulate debris, and the resulting supernatant was sequentially filtered twice through three layers of sterile gauze. The final microbial suspension was administered to recipient rats via intragastric gavage within 30 min of preparation.

All animal experiments were reported in accordance with the ARRIVE guidelines. The completed ARRIVE 2.0 checklist is provided as a supplementary file.

### In vitro assays

IEC-18 cells (Cellverse Co., Ltd., Catalog No. iCell-r015, Shanghai, China) were cultured according to the instructions. During the intervention, the cells were exposed to media containing 50 μM THDCA and 50 μM HDCA for 48 h. The primary antibody after undergoing a series of treatments was added, and the cells were incubated overnight at 4 °C, followed by five washes with PBS for 3 min each. The corresponding antibody was applied and incubated in the dark for 1 h, followed by six washes with PBS for 3 min each. Nuclei were stained with DAPI for 10 min and washed three times with PBS, each for 3 min. Finally, 500 µL of PBS was added to each well in preparation for imaging, which was performed using an inverted fluorescence microscope.

### Analytical methods

#### Measurement of BSH activity

BSH activity was measured in homogenates prepared from fresh ileal luminal contents. This assay is based on the enzymatic hydrolysis of conjugated bile acids (e.g., taurocholic or glycocholic acid) by BSH, which liberates free bile acids and amino acids (taurine or glycine). The released amino acids react with ninhydrin reagent to form a purple complex, the absorbance of which is directly proportional to the amino acid concentration. Consequently, BSH activity was indirectly quantified by measuring the production of glycine or taurine. Fresh ileal luminal contents (500 mg) were rinsed with ice-cold PBS to remove debris and homogenized in 50 mM PBS (pH 7.0) at a 1:10 (w/v) ratio. Homogenates were centrifuged at 12,000 × g for 10 min at 4 °C, and the resulting supernatants were collected as the enzyme source. Protein concentration was determined using the Bradford assay. The reaction mixture (200 μL final volume) consisted of 100 μL of tissue supernatant and substrate solution containing either 5 mM taurocholic acid (TCA) or 5 mM glycocholic acid (GCA) in 50 mM PBS (pH 7.0). Reactions were incubated at 37 °C for 30 min to 2 h. A negative control lacking the enzyme source was included to correct for non-enzymatic hydrolysis. Reactions were terminated by the addition of an equal volume of 10% (w/v) trichloroacetic acid, followed by centrifugation at 12,000 × g for 10 min at 4 °C. For colorimetric detection, 200 μL of the reaction supernatant was mixed with 200 μL of ninhydrin reagent (0.25 g ninhydrin dissolved in 5 mL of 70% acetic acid, adjusted to 100 mL with distilled water). The mixture was heated in a boiling water bath (100 °C) for 10 min, cooled to room temperature, and adjusted to a final volume of 1 mL with distilled water. Absorbance was measured at 570 nm. Standard curves were generated using glycine or taurine solutions (0-200 μM) treated identically to the samples. All determined BSH activities were normalized against total protein content of each sample homogenate. BSH activity was calculated based on the released amino acids and expressed as U/mg protein using the formula: Activity (U/mg) = Product (nmol)/Time (min) × Protein (mg). BSH activity against GCA and TCA substrates was determined respectively. BSH activity in fecal samples was also determined using the same assay.

#### Biochemical analysis

For human studies, serum lipid profiles and other biochemical parameters were measured using a Cobas 8000 modular analyzer (Roche Diagnostics, Shanghai, China) according to the manufacturer’s instructions. For animal experiments, serum inflammatory cytokines were quantified using ELISA kits (Nanjing Laputa Biotechnology Co., Ltd., Nanjing, China), whereas serum lipid profiles were determined using commercial assay kits from Nanjing Jiancheng Bioengineering Institute (Nanjing, China).

#### Histopathological examination

Liver tissues were harvested and fixed in 4% paraformaldehyde following standard protocols, then embedded in paraffin. Tissue sections were stained with hematoxylin and eosin (H&E) for histological examination. The severity of hepatic lesions, hepatocellular steatosis, and inflammation was assessed using the corresponding established criteria.

#### Intestine immunofluorescence staining analysis

Intestinal tissues were fixed in 4% paraformaldehyde at 4 °C for 6 h, followed by dehydration in sequential 15% and 30% sucrose solutions for 12 h each. Continuous sections of 5 μm thickness were cut and adhered to poly-L-lysine coated slides, stored at −20 °C for future use. Before staining, sections were allowed to reach room temperature and gently washed with PBS three times for 5 min each. They were permeabilized with 0.2% Triton X-100 for 10 min at room temperature, followed by blocking with 5% BSA for 30 min at room temperature. Before staining, sections were allowed to reach room temperature and gently washed with PBS three times for 5 min each. They were permeabilized with 0.2% Triton X-100 for 10 min at room temperature, followed by blocking with 5% BSA for 30 min at room temperature. The primary antibody (FXR, orb156973, Biorbyt; diluted 1:200 in 1% BSA-PBS) was added and incubated overnight in a humid chamber at 4 °C. The next day, slides were washed three times with PBS for 5 min each and then stained with Alexa Fluor 488 goat anti-rabbit IgG (1:500) in the dark at room temperature for 1 h. Following PBS washes, nuclei were stained with DAPI (1 μg/mL) for 5 min, and coverslips were mounted with buffered glycerol. Images were captured using a Nikon DS-Ri2 digital microscope, maintaining consistent exposure parameters, and fluorescence intensity was quantified using ImageJ software (version 1.54).

#### Measurement of BA

Detailed protocols for BA quantification have been described in our previous study.^14^ Briefly, 25 mg of the sample was weighed and mixed with 1,000 μL of extraction solvent composed of methanol, acetonitrile, and water in a ratio of 2:2:1 (v/v/v), along with isotope-labeled internal standards. The mixture was vortexed for 30 s, homogenized using steel beads at 35 Hz for 4 min, and sonicated in an ice-water bath for 5 min. This grinding and sonication process was repeated three times. The samples were then incubated at −40 °C for 1 h to precipitate proteins. After centrifugation at 12,000 rpm for 15 min at 4 °C, the supernatants were transferred to LC vials for analysis. Chromatographic separation was performed using a Vanquish UHPLC system (Thermo Fisher Scientific) equipped with a Waters ACQUITY UPLC BEH C18 column (150 × 2.1 mm, 1.7 μm). Mass spectrometric detection was conducted using an Orbitrap Exploris 120 high-resolution mass spectrometer (Thermo Fisher Scientific) operating in Parallel Reaction Monitoring mode. The raw data were processed and quantified using the appropriate software.

#### Microbial diversity 16 s rRNA gene amplicon sequencing

Total genomic DNA was extracted from 0.25−0.5 g of fecal samples using a magnetic bead-based method. The samples were placed in 2 mL centrifuge tubes containing 500 μL of Buffer SA, 100 μL of Buffer SC, 0.25 g of grinding beads, and 10 μL of RNase A. The mixture was homogenized by vortexing or with a tissue homogenizer and lysed at 70 °C for 15 min. Following centrifugation at 12,000 rpm for 1 min, the supernatant (~500 μL) was transferred to a new tube, mixed with 200 μL of Buffer SH, and incubated at 4 °C for 10 min. Following centrifugation at 12,000 rpm for 3 min, the supernatant was mixed with 500 μL Buffer GFA and 10 μL Magnetic Bead Suspension G, then incubated for 5 min to allow binding. The beads were captured using a magnetic stand for 30 s, and the supernatant was discarded. The beads were washed sequentially with 700 μL Buffer RD for 5 min and then with 700 μL Buffer PWD twice for 3 min each. After air-drying for 5−10 min, DNA was eluted in 50−100 μL Elution Buffer TB at 56 °C for 5 min with intermittent shaking. The purified DNA was transferred to a 96-well plate and stored under appropriate conditions. DNA concentration was quantified using a 1 × dsDNA HS Working Solution on a microplate reader. The integrity of PCR products was verified by 1.8% agarose gel electrophoresis or a LabChip GX Touch system. For human fecal samples, the V3-V4 hypervariable regions of the bacterial 16S rRNA gene were amplified. For animal fecal samples, full-length 16S rRNA genes were amplified using specific primers tagged with 8-bp barcodes at the 5′ end for multiplexing. Successful amplicons were purified using magnetic beads, subjected to a s round of PCR, and pooled to generate the final sequencing libraries. High-quality sequences were obtained after quality assessment. Alpha and beta diversity indices were calculated to evaluate microbial diversity. Community structure and differential abundance analyzes were conducted based on taxonomic classification.

#### Western blot analysis

Liver and ileal tissues were lysed in ice-cold RIPA buffer (Beyotime Biotechnology, Shanghai, China) and centrifuged at 14,000 × g for 5 min. Supernatants were collected, and total protein concentrations were determined using a BCA Protein Assay Kit (Thermo Fisher Scientific, USA). Protein extracts were adjusted to 5 μg/μL, mixed with loading buffer (Beyotime Biotechnology), and denatured by boiling at 100 °C for 10 min. Equal amounts of protein were resolved by 10% SDS-PAGE and transferred to PVDF membranes. Membranes were blocked with 5% non-fat milk and subsequently incubated with the following primary antibodies: CYP7A1 (1:1000, A22897, RRID:AB_3105781, ABclonal), CYP8B1 (1:1000, A25847, RRID:N/A, ABclonal), CYP27A1 (1:5000, A23250, RRID:AB_3674587, ABclonal), CYP7B1 (1:1000, A17872, RRID:AB_2769104, ABclonal), FXR (1:2000, A24015, RRID:N/A, ABclonal), SHP (1:1000, A16454; RRID:AB_2770666, ABclonal), FGF19 (1:500, A6589; RRID:AB_2767182, ABclonal), *β*-actin (1:15000, GB11001-100, RRID:N/A, Servicebio) at 4 °C overnight. Notably, rodents express FGF15 rather than human FGF19. Human FGF19 and rat FGF15 are orthologous proteins sharing extensive sequence homology. The anti-FGF19 polyclonal antibody (A6589) was generated against a recombinant human FGF19 fragment spanning amino acids 25-216. Sequence alignment identifies several stretches of identical residues within this region; linear epitopes typically consist of 5-8 continuous amino acids, and polyclonal antibodies recognize multiple distinct epitopes. Based on these conserved sequence motifs, this antibody is predicted to cross-react with rat FGF15 orthologs. The theoretical molecular weight of human FGF19 is 24 kDa, and the target band corresponding to rat FGF15 was consistently detected at approximately 22 kDa in our ileal tissue blots. Membranes were washed three times with TBST (Tris-buffered saline with Tween 20) and incubated with Goat anti-Rabbit IgG (H+L) (1:15000, Cat# G1213, Servicebio) for 2 h. Following a final wash step, protein bands were visualized using an ECL kit (Bio-Rad, Hercules, CA, USA).

### Statistical analysis

#### Clinical and experimental data

The normality of continuous variables was assessed using Q-Q plots followed by the Shapiro-Wilk test. Normally distributed continuous variables are expressed as mean ± standard deviation (SD) and were compared using Student's t tests or analysis of variance (ANOVA). Non-normally distributed variables are expressed as median with interquartile range (IQR) and were compared using the Mann-Whitney U test or Kruskal-Wallis H test. Categorical variables are presented as frequencies (percentages) and were compared using Pearson's χ^2^ test or Fisher's exact test. Binary logistic regression was performed to evaluate the association between PS intake levels and dyslipidemia. Three adjusted models were established to calculate Odds Ratios (ORs) and 95% Confidence Intervals (95% CIs). Furthermore, restricted cubic splines (RCS) were used to examine potential nonlinear associations between dietary PS intake and the risk of dyslipidemia. Generalized Estimating Equations (GEE) were used to compare primary and secondary outcomes between groups for clinical trial 1. The GEE models included group, time (0 and 3 months), and the group-by-time interaction as independent variables. The models specified a linear link function, a robust estimator for the covariance matrix, and an exchangeable working correlation matrix. When a significant group-by-time interaction was observed, pairwise comparisons were performed to evaluate differences between intervention groups. No multiplicity adjustment was applied to these prespecified pairwise contrasts. Multiple imputation was performed using Multivariate Imputation by Chained Equations (MICE) under the missing completely at random (MCAR) assumption, generating five imputed datasets. All analytical variables were incorporated into the multiple imputation model, including waist circumference, blood pressure, body weight, height, marital status, smoking status, drinking status, ethnicity, educational level, and physical activity level. Predictive mean matching was adopted for continuous variables, logistic regression for binary variables, and multinomial logistic regression for unordered categorical variables. Diagnostic checks were performed for each imputation model to assess convergence and the plausibility of imputed values. Separate statistical models were fitted on each completed dataset, and pooled effect estimates were synthesized following Rubin’s rules. All participants were included in the Intention-to-Treat (ITT) analysis. The Per-Protocol (PP) analysis included only participants who adhered to the study protocol. Primary results were reported based on the ITT analysis, while sensitivity analyzes using the PP population were conducted to verify the robustness of the primary and secondary outcomes.

For animal and in vitro experiments, data are expressed as mean ± standard error of the mean (SEM). For comparisons between two groups, normally distributed data were analyzed using unpaired two-sided Student's t tests, while non-normally distributed data were analyzed using the Mann-Whitney U test. For comparisons involving multiple groups, one-way ANOVA was performed for normally distributed variables. Fisher’s least significant difference (LSD) test was used exclusively for a priori specified pairwise comparisons that were defined before data analysis and was not applied as a general exploratory post hoc test. Non-normally distributed data were assessed via the Kruskal-Wallis H test, with Dunn’s test applied for pairwise comparisons. Quantification of Western blot band gray intensity was performed with ImageJ software. The relative protein expression level was calculated as the gray value ratio of target protein to the corresponding internal reference protein (e.g., β‑actin). Spearman correlation analysis was performed to evaluate the associations between BA and metabolic parameters.

#### Multi-omics data

For gut microbiota data from the human intervention trial, before log-ratio transformation, zero counts were handled using Bayesian multiplicative replacement to permit log-ratio analysis while preserving the relative structure among non-zero components. Centered log-ratio (CLR) transformation was subsequently applied. CLR-normalized data were fitted into linear mixed-effects models (LMM) to quantify intervention-induced shifts in CLR-transformed taxon abundance. Model coefficients (*β*) represented adjusted effects on the CLR scale, whereas estimated marginal means and pairwise contrasts were obtained using the *emmeans* package (v.2.0.1). For animal-derived gut microbiota datasets, community structure was profiled via alpha diversity metrics and bray-curtis-based beta diversity. Alpha-diversity differences were assessed using the Kruskal-Wallis H test followed by Dunn’s test for pairwise comparisons. Beta diversity was calculated using Bray-Curtis dissimilarity, visualized by principal coordinates analysis (PCoA), and group differences in community composition were assessed using PERMANOVA. At the taxonomic composition level, linear discriminant analysis effect size (LEfSe) was used to identify differentially abundant taxa, with an LDA score > 4.0 used as the effect-size threshold. Non‑parametric Kruskal-Wallis test followed by Dunn’s test was adopted for intergroup comparisons of gut microbial taxa.

For serum BA data from the human intervention trial, all raw concentrations (nmol/L) were ln(x + 1)-transformed before GEE analysis to reduce skewness and stabilize variance. GEE was used to compare BA profiles at baseline and month 3, with group, time and the group-by-time interaction set as predictors. The model adopted a linear link, robust covariance estimation and exchangeable working correlation, adjusted for age, sex and baseline BMI. Prespecified pairwise contrasts were evaluated without multiplicity adjustment and were restricted to comparisons defined before data analysis. Original log-scale regression coefficients (*β*) from GEE were exponentiated. For animal BA data, two-group comparisons were performed using the Mann-Whitney U test, whereas comparisons involving more than two groups were performed using the Kruskal-Wallis H test followed by Dunn’s test for pairwise comparisons. Spearman’s rank correlation analysis was performed to explore associations among BAs, altered gut microbiota, and host lipid phenotypes. Given the exploratory nature of the BA screening and correlation analyzes, no multiplicity correction was applied. All statistical analyzes were performed using IBM SPSS Statistics version 30.0 (SPSS Inc., Chicago, IL, USA) and R software (version 4.3.2; http://www.R-project.org). For prespecified analyzes, a two-sided *P* value < 0.05 was considered statistically significant. For exploratory BA screening and correlation analyzes, nominal *P* values are reported and were interpreted as exploratory rather than confirmatory.

## Results

### Dietary phytosterol intake is associated with blood lipid profiles in a human cross-sectional study

**Figure 1.**
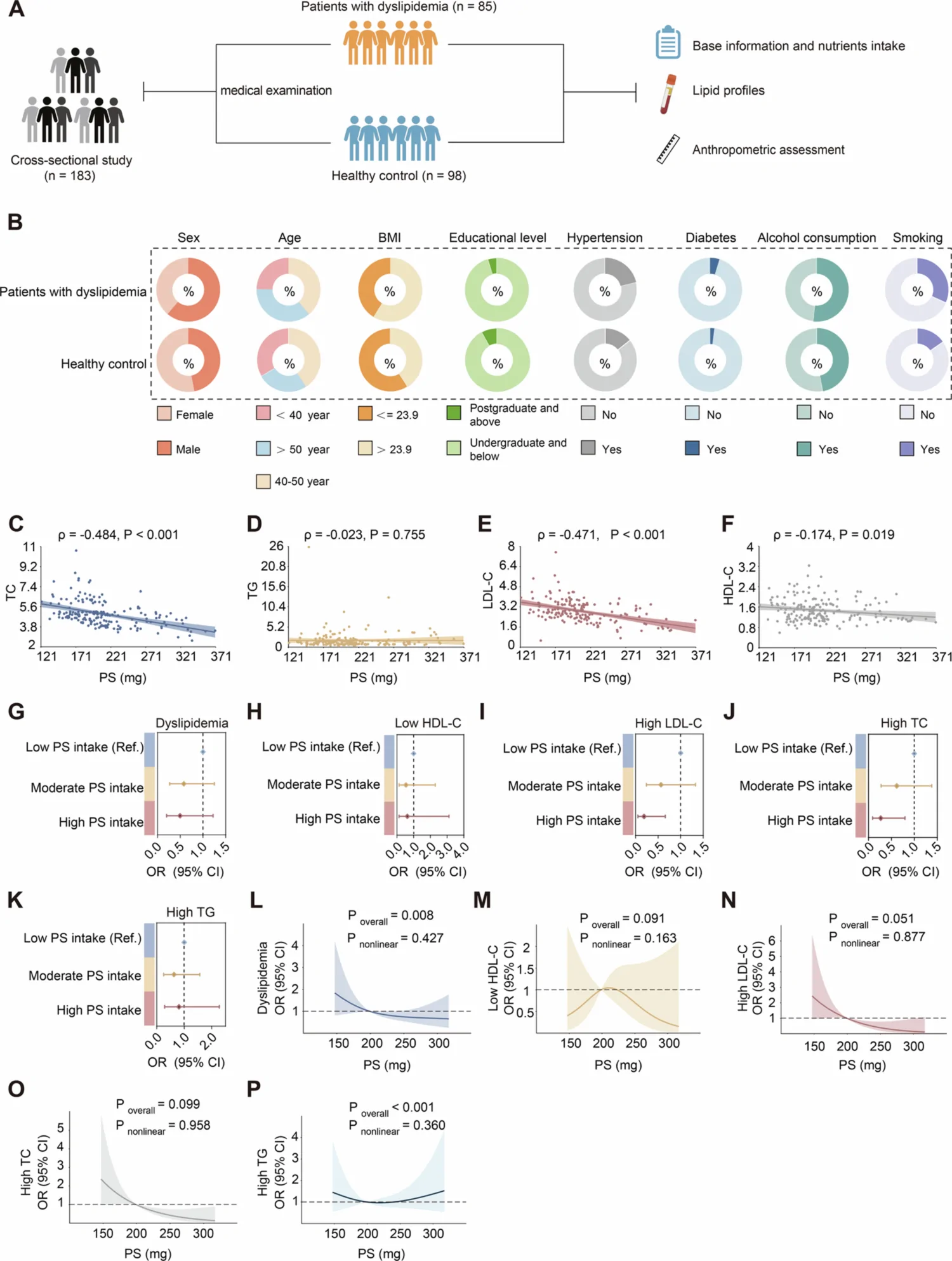
Dietary phytosterol intake is associated with blood lipid profiles in a human cross-sectional study. (**A**) Study overview. Participants were recruited from residential communities in the Gulou District, Nanjing, China. Fasting blood samples were collected for measurement of serum lipid profiles. Dietary intake was assessed using a validated food frequency questionnaire, and total daily phytosterol (PS) intake was estimated using nutrient composition data from the Sixth Edition of the Chinese Food Composition Table. (**B**) Donut charts showing the distribution of selected baseline characteristics across dietary PS intake groups. (**C–F**) Spearman correlation analyzes between dietary PS intake and serum TC, TG, LDL-C, and HDL-C, respectively. *ρ* denotes Spearman’s rank correlation coefficient. (**G–K**) Forest plots showing adjusted ORs and 95% CIs for the associations of dietary PS intake categories with the risk of dyslipidemia, low HDL-C, high LDL-C, high TC, and high TG, respectively. Multivariable logistic regression models were adjusted for age, sex, BMI, smoking status, alcohol consumption, and total physical activity. The lowest PS intake category was used as the reference group (Ref). (**L–P**) Restricted cubic spline analyzes illustrating the associations between dietary PS intake and the risk of dyslipidemia, low HDL-C, high LDL-C, high TC, and high TG, respectively. Models were fitted using three knots located at the 10th, 50th, and 90th percentiles of the PS intake distribution, selected based on the Akaike information criterion (AIC). Solid lines represent adjusted ORs, and shaded areas indicate the corresponding 95% CIs. Models were adjusted for age, sex, BMI, smoking status, alcohol consumption, and total physical activity.

To investigate the association between dietary PS and plasma lipid profiles, we conducted a cross-sectional study involving 183 participants, comprising 85 individuals with dyslipidemia and 98 healthy controls. Our study design is summarized in Figure 1A. The dyslipidemia group differed significantly from healthy controls in mean age, BMI categories, and smoking status (Figure 1B and Table S4). Individuals aged 40–50 y accounted for the largest proportion (39.9%), and the proportion of males was slightly higher than that of females (53.6% vs. 46.4%). Most had an education level of undergraduate or below (93.4%). Based on BMI classification, 50.8% had a BMI ≤ 23.9, and 49.2% had a BMI > 23.9. Regarding lifestyle factors, 23.0% were smokers and 49.2% reported alcohol consumption. No significant differences were found between groups in total PS intake (226.99 vs. 232.84 mg/d), total energy intake, or physical activity level (all *P* > 0.05) (Table S4). Figure 1C–F illustrate the associations between total PS intake and serum lipid profiles using scatter plots with Spearman’s correlation regression lines. Total PS intake was moderately and inversely correlated with TC and LDL-C levels (both *P* < 0.001), and weakly inversely correlated with HDL-C levels (*P* = 0.019) but showed no significant correlation with TG (*P* > 0.05). Subsequently, binary logistic regression models were utilized to determine the association of total PS intake with dyslipidemia and its specific phenotypes. After adjustment for potential confounders, no significant associations were observed between total PS intake and risk of overall dyslipidemia, low HDL-C, or high TG (all *P*_trend_ > 0.05). In contrast, high PS intake was significantly associated with lower risk of high LDL-C (OR = 0.188; 95% CI: 0.053-0.661) and high TC (OR = 0.267; 95% CI: 0.090-0.795), with both *P*_trend_ < 0.05 (Figure 1G–K). We also used RCS models to examine potential non-linear associations between total PS intake and risk of dyslipidemia and its subtypes. No significant non-linear relationships were observed between PS intake and risk of overall dyslipidemia, low HDL-C, high LDL-C, high TC, or high TG (all *P* > 0.05). However, PS intake was inversely associated with risk of overall dyslipidemia (*P* = 0.008), while inverse but non-significant trends were observed for high LDL-C and high TC (*P* > 0.05) (Figure 1L–P). Collectively, these data point to a consistent association between dietary PS intake and circulating lipid profiles, offering an epidemiological foundation to inform subsequent interventional mechanistic studies.

### High-phytosterol diet intervention modulates lipid profiles and is accompanied by alterations in gut microbiota and serum BAs

**Figure 2.**
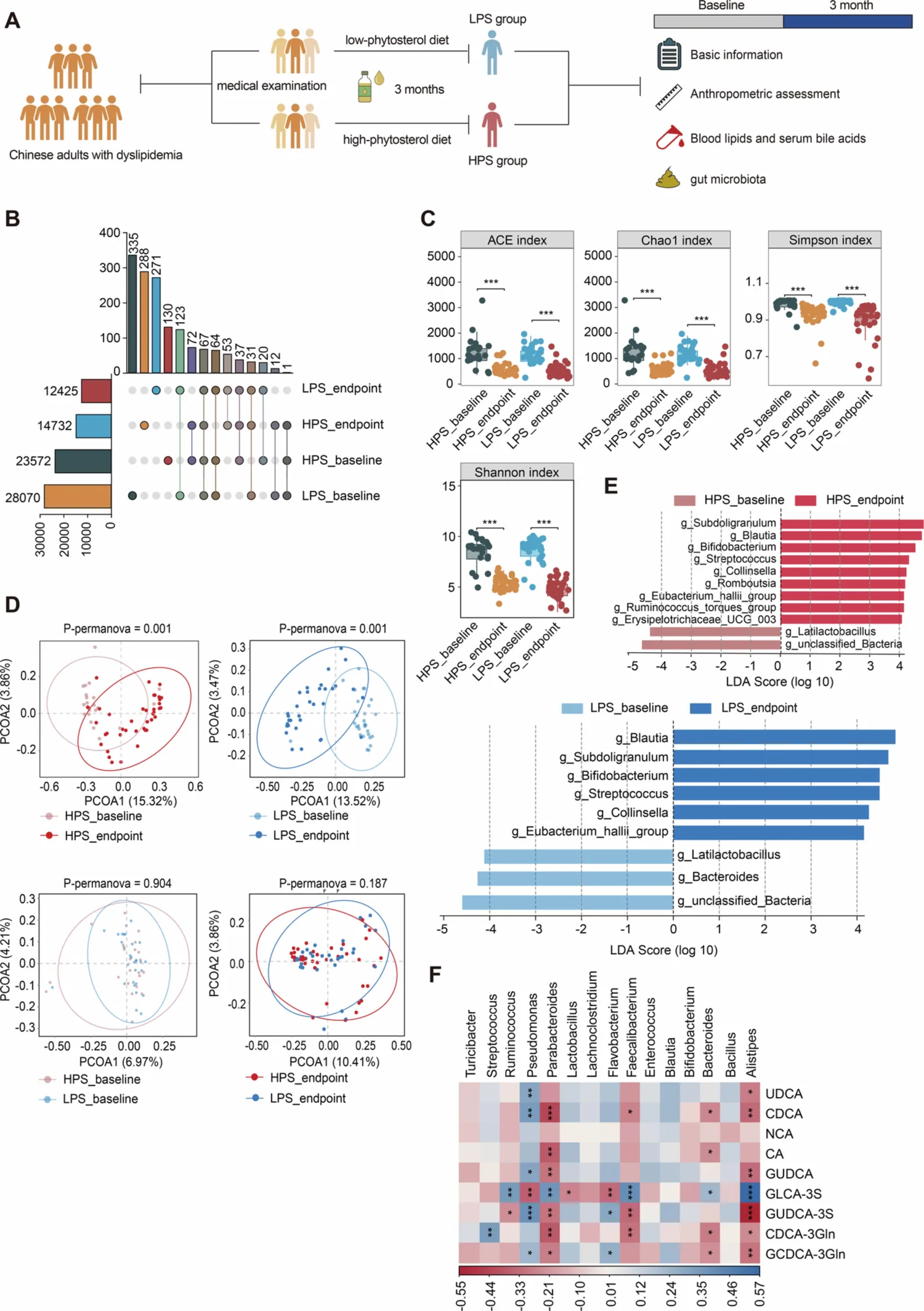
High-phytosterol diet intervention modulates lipid profiles and is accompanied by alterations in gut microbiota and serum BAs. (**A**) Schematic overview of the randomized controlled dietary intervention. A total of 104 participants with dyslipidemia were randomized to the low-phytosterol diet (LPS) or high-phytosterol diet (HPS) group (*n* = 52 randomized participants/group) for 3 months. Participants in the LPS group used low-PS peanut oil as their primary cooking oil, whereas those in the HPS group used high-PS blended oil composed of corn germ oil and wheat germ oil. Both groups received standardized, identical lunches provided by the study, while reference menus were provided for breakfast and dinner, allowing participants to make their own food choices within the recommended dietary framework. (**B**) UpSet plot showing shared and unique OTUs across the four group-by-time sample sets at baseline and after 3 months of intervention. A total of 124 fecal samples were available for microbiome analysis (LPS_baseline, *n* = 30; LPS_endpoint, *n* = 33; HPS_baseline, *n* = 22; HPS_endpoint, *n* = 39). The lower-left bar chart indicates the total number of OTUs in each sample set, whereas the upper bar chart indicates the number of OTUs corresponding to each intersection. Dots and connecting lines in the matrix indicate the sample sets contributing to each intersection. (**C**) Alpha-diversity comparisons across groups and time points. Overall differences were assessed using the Kruskal-Wallis H test, followed by Dunn’s test for pairwise comparisons. *P < 0.05; **P < 0.01; ***P < 0.001. (**D**) Beta-diversity analysis of gut microbial community composition across groups and time points. Differences in community composition were assessed using permutational multivariate analysis of variance (PERMANOVA). (**E**) Genus-level microbial taxa differing across the specified group comparisons were identified using linear discriminant analysis effect size (LEfSe). An LDA score > 4.0 was used as the effect-size threshold. **(F)** Spearman correlation heatmap showing associations between putative BSH-producing taxa and candidate serum BAs at the study endpoint. *P < 0.05; **P < 0.01; ***P < 0.001.

To further investigate the effect of PS-enriched diet on blood lipids, BA metabolism, and gut microbiota among adults with dyslipidemia, we conducted a three-month, outcome assessor-blinded, randomized controlled feeding trial among Chinese adults with dyslipidemia. The study design is summarized in Figure 2A. Our previous study has established the lipid-lowering efficacy of a habitually high-PS diet, demonstrating that habitually high dietary PS intake significantly reduced serum TC, TG, LDL-C and Non-HDL-C levels compared to a low-PS regimen.^11^ To investigate the gut microbiota alterations, we performed 16S rRNA gene amplicon sequencing on collected fecal samples. Operational Taxonomic Unit (OTU) clustering of all samples across baseline and endpoint yielded a total of 94,136 OTUs from 7,380,889 paired-end reads, with 67 core OTUs shared across all four subgroups (Figure 2B). At baseline, no significant differences were observed in either alpha or beta diversity between groups, indicating comparable initial microbial profiles. We found that alpha diversity differed significantly between baseline and 3 months in both dietary groups after 3-months intervention (Figure 2C). For adaptations of the gut microbiome during the intervention, a significant difference between baseline and endpoint (3 months) in beta diversity was found in the HPS diet and LPS diet groups (*P*_PERMANOVA_ < 0.05, Figure 2D). The PS dietary intervention induced significant alterations in the taxonomic profile of the gut microbiota, characterized by distinct remodeling patterns between the two groups. We further characterized the microbial taxonomic composition by profiling the relative abundances of the 12 most abundant phyla and genera (Figure S1). Following the 3-month intervention, phylum-level analysis revealed an expansion of *Firmicutes* in the HPS diet group, concomitant with a contraction in the abundance of *Bacteroidetes* and *Proteobacteria*. At the genus level, we observed an enrichment of several gut microbiota in the HPS diet group, including *Bacteroides*, *Escherichia-Shigella*, *Romboutsia*, *Blautia*, *Bifidobacterium*, *Faecalibacterium*, *Streptococcus*, and *Collinsella*. In contrast, the relative abundance of *Coprobacillus* was notably reduced. At the phylum level, no significant differences in relative abundance were observed between two groups at the endpoint (*P* > 0.05). A uniform depletion of *Latilactobacillus* was observed in both groups after the intervention (Figure 2E). To further identify genera specifically responsive to the HPS diet, LMM analysis was performed using CLR-transformed genus-level abundance data after adjustment for age, sex, and BMI. Several genera showed significant group-by-time interaction effects (Table S5). On the CLR scale, *Defluviitoga* showed a positive LS mean difference (*P* < 0.05), whereas the remaining responsive genera showed negative LS mean differences (*P* < 0.05), including *Arenimonas*, *Cetobacterium*, *Opitutus*, *Dietzia*, *Limosilactobacillus*, and *Mycoplasma*. Given the role of BSH-active bacteria in bile acid metabolism, we further examined 15 literature-reported BSH-active genera. Although no genus yielded a statistically significant group-by-time interaction (*P* > 0.05), 13 out of the 15 assessed genera displayed negative least-squares mean differences, indicative of a potential declining trend in putative BSH-producing taxa upon high-phytosterol dietary intervention (Table S6).

To assess the impact of HPS and LPS dietary regimens on BA metabolism, serum BA profiles were quantified using a targeted UHPLC-PRM-MS/MS approach. In GEE models, significant group-by-time interaction effects were observed for the primary BAs chenodeoxycholic acid (CDCA) and cholic acid (CA) in both unadjusted and adjusted models (*P* < 0.05). After adjustment for age, sex, and baseline BMI, CDCA and CA were significantly decreased from baseline in the HPS group, but not in the LPS control group. At 3 months, the HPS group showed significantly lower serum levels of CDCA and CA than the LPS group (Tables S7 and S8). In addition to free primary BAs, several conjugated or modified BAs also differed between groups after intervention. Compared with the LPS group, the HPS group had lower serum levels of chenodeoxycholic acid-3-*β*-D-glucuronide (CDCA-3Gln) and glycochenodeoxycholic acid-3-O-*β*-glucuronide (GCDCA-3Gln) (Table S8). Among secondary BAs, glycolithocholic acid-3-Sulfate (GLCA-3S) showed a significant group-by-time interaction after covariate adjustment (*P* < 0.05). At 3 months, the HPS group exhibited significantly lower levels of ursodeoxycholic acid (UDCA), nor cholic acid (NCA), glycoursodeoxycholic acid (GUDCA) and glycoursodeoxycholic acid-3-sulfate (GUDCA-3S) than the LPS group (Tables S9 and S10). Collectively, these findings indicate that the HPS diet intervention reshaped the circulating bile acid pool, particularly free primary BAs and several conjugated or modified BA species, suggesting that BA remodeling may be involved in the regulation of enterohepatic circulation.

To explore the associations among the gut microbiota, serum BA, and clinical lipid indices, spearman correlation analysis was performed. Intervention-related changes in several candidate BAs were significantly associated with changes in serum lipid parameters. In particular, changes in CDCA were positively correlated with changes in TG, non-HDL-C, and ApoB, but negatively correlated with HDL-C, whereas changes in GLCA-3S were negatively correlated with TG. Several conjugated or modified BA species, including GUDCA-3S, CDCA-3Gln, and GCDCA-3Gln, were positively correlated with TG changes, and GCDCA-3Gln was inversely associated with HDL-C and ApoA1 (Figure S2). Changes in several putative BSH-producing taxa were also associated with lipid-related indices. *Bacteroides*, *Bifidobacterium*, *Faecalibacterium*, and *Parabacteroides* were negatively correlated with TG changes, whereas *Flavobacterium*, *Lachnoclostridium*, and *Lactobacillus* were positively correlated with TG changes. In addition, *Faecalibacterium* was positively associated with HDL-C, while *Flavobacterium* was negatively associated with both HDL-C and ApoA1 (Figure S3). We further observed broad correlations between putative BSH-producing taxa and candidate BAs. *Alistipes*, *Bacteroides*, *Faecalibacterium*, and *Parabacteroides* were negatively correlated with multiple BA species, particularly CDCA, CA, GUDCA-3S, CDCA-3Gln, and GCDCA-3Gln, whereas *Alistipes* and *Faecalibacterium* were positively correlated with GLCA-3S. In contrast, *Pseudomonas* was positively associated with several BAs, including UDCA, CDCA, GUDCA, GUDCA-3S, and GCDCA-3Gln (Figure 2F). Collectively, HPS dietary intervention was associated with improved lipid profiles and coordinated alterations in gut microbiota and circulating BAs. These findings suggest that putative BSH-producing taxa-related BA remodeling may contribute to lipid metabolic changes. Given the distinct fatty acid compositions of the HPS and LPS oil matrices, subsequent animal experiments using purified PS were performed to further verify the specific lipid-regulatory effects of PS and related gut microbiota-BA mechanisms.

### Phytosterols improve serum lipid profiles and attenuate inflammatory responses in HFD-fed rats

**Figure 3.**
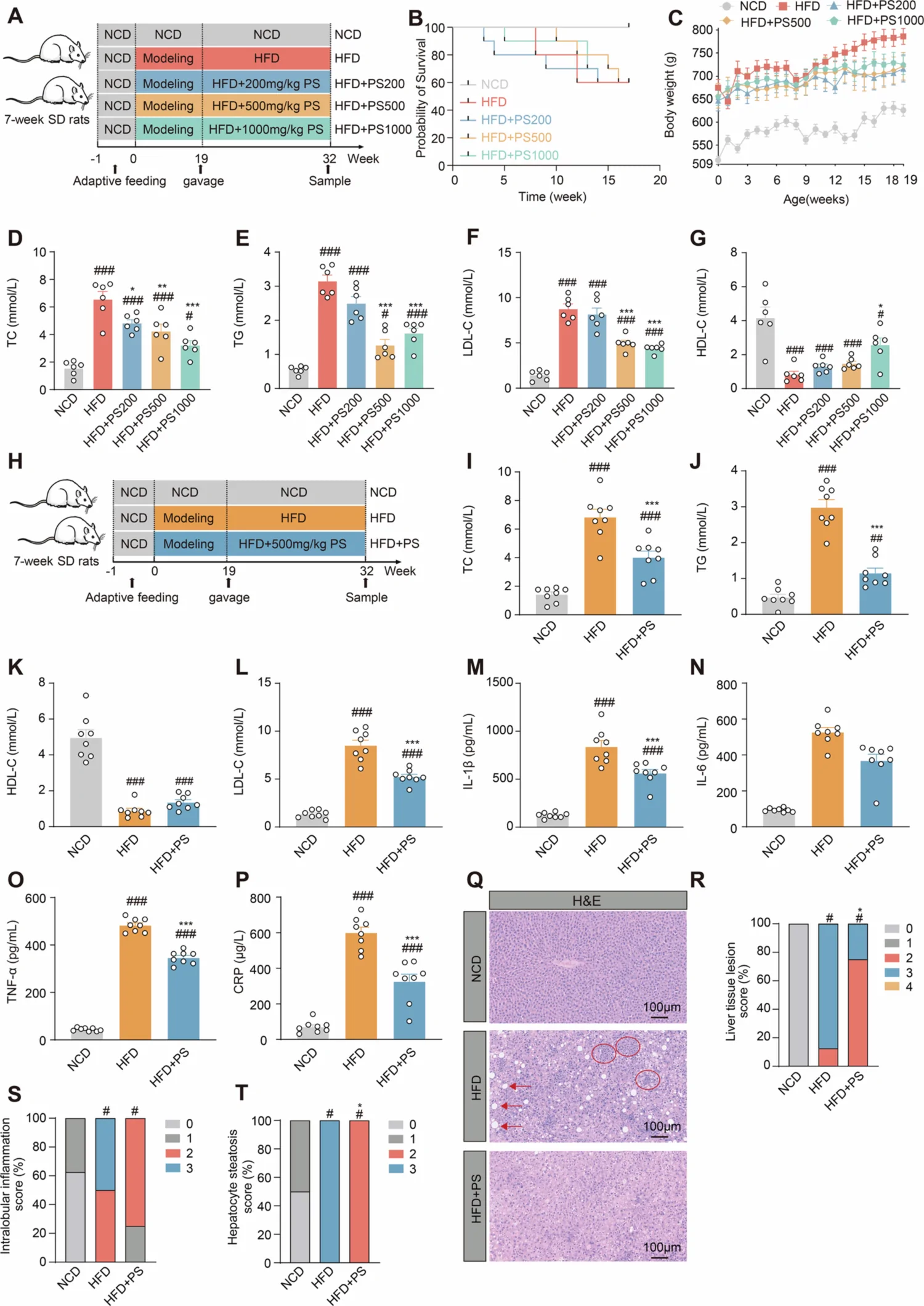
Phytosterols improve serum lipid profiles and attenuate inflammatory responses in HFD-fed rats. (**A**) Experimental groups: NCD, normal control diet; HFD, high-fat diet; HFD+PS200, HFD supplemented with 200 mg/kg phytosterols; HFD+PS500, HFD supplemented with 500 mg/kg phytosterols; and HFD+PS1000, HFD supplemented with 1000 mg/kg phytosterols. Ten animals were initially included in each group for survival analysis. Following mortality during the intervention period, six surviving animals per group were selected for subsequent biochemical analyzes. (**B**) Kaplan-Meier survival curves for the five experimental groups. Each group initially comprised 10 animals. Overall differences in survival among groups were assessed using the log-rank test. When the overall test was statistically significant, pairwise log-rank comparisons were subsequently performed. (**C**) Changes in body weight over time in each experimental group during the intervention period. (**D–G**) Serum levels of TC, TG, LDL-C and HDL-C in each group after 13 weeks of intervention, respectively (*n* = 6 animals/group). Data are presented as mean ± SEM. Overall group differences were assessed by one-way ANOVA, with Fisher’s LSD test applied exclusively to pairwise comparisons specified a priori. ^#^ compared to NCD, *P* < 0.05; ^##^ compared to NCD, *P* < 0.01; ^###^ compared to NCD, *P* < 0.001. (**H**) Experimental groups: NCD, HFD, and HFD+PS (HFD supplemented with 500 mg/kg phytosterols; *n* = 8 animals/group). (**I–L**) Serum levels of TC, TG, LDL-C and HDL-C in each group after 13 weeks of intervention, respectively (*n* = 8 animals/group). Data are presented as mean ± SEM. Overall group differences were assessed by one-way ANOVA, with Fisher’s LSD test applied exclusively to pairwise comparisons specified a priori. ^#^ compared to NCD, *P* < 0.05; ^##^ compared to NCD, *P* < 0.01; ^###^ compared to NCD, *P* < 0.001. * compared to HFD, *P* < 0.05; ** compared to HFD, *P* < 0.01; *** compared to HFD, *P* < 0.001. (**M–P**) Serum levels of IL-1β, IL-6, TNF-*α* and CRP in each group after 13 weeks of intervention, respectively (*n* = 8 animals/group). Data are presented as mean ± SEM. Overall group differences were assessed by one-way ANOVA, with Fisher’s LSD test applied exclusively to pairwise comparisons specified a priori. ^#^ compared to NCD, *P* < 0.05; ^##^ compared to NCD, *P* < 0.01; ^###^ compared to NCD, *P* < 0.001. * compared to HFD, *P* < 0.05; ** compared to HFD, *P* < 0.01; *** compared to HFD, *P* < 0.001. (**Q**) Representative hematoxylin and eosin (H&E)-stained liver sections. Scale bar, 100 μm. Red ellipses indicate inflammatory cell infiltration, and red arrows indicate lipid vacuoles. **(R–T)** Distributions of liver lesion scores (R), intralobular inflammation scores (S), and hepatic steatosis scores (T). Liver lesions were scored on a 0–4 scale, whereas intralobular inflammation and hepatic steatosis were scored on 0–3 scales, with higher scores indicating greater histopathological severity. Overall differences among groups were assessed using the Kruskal-Wallis H test, followed by Dunn's test for pairwise comparisons. ^#^ compared to NCD, *P* < 0.05; ^##^ compared to NCD, *P* < 0.01; ^###^ compared to NCD, *P* < 0.001. * compared to HFD, *P* < 0.05; ** compared to HFD, *P* < 0.01; *** compared to HFD, *P* < 0.001.

Our clinical data indicated that the HPS diet ameliorates lipid metabolism, a benefit linked to alterations in the gut microbiota and BA profiles. Based on these observations, we hypothesized that the hypolipidemic efficacy of PS derived from wheat and corn germ blended oil is mediated by the gut microbiota-BA axis. To elucidate the underlying mechanisms, we conducted further investigations using a hyperlipidemic rat model. PS were administered at a dosage of 500 mg/kg/day, which was determined based on our preliminary studies (animal experiment 1, Figure 3A–G). The experimental design for the optimal dose PS intervention is presented in Figure 3H. The results showed that 500 mg/kg/day PS significantly reduced serum TC, TG, and LDL-C levels (*P* < 0.05) (Figure 3I–L). Furthermore, we observed that PS markedly lowered the levels of serum pro-inflammatory cytokines, including IL-1β, TNF-*α*, and CRP levels (*P* < 0.05). Although IL-6 levels also showed a downward trend following PS treatment, the change did not reach statistical significance (*P* > 0.05) (Figure 3M–P). HE staining of rat liver tissues are as shown in Figure 3Q. The HFD group showed apparent hepatic steatosis characterized by numerous lipid vacuoles and inflammatory cell infiltration compared with NCD group. However, PS intervention markedly attenuated hepatic histopathological abnormalities, as evidenced by reduced lipid accumulation and alleviated inflammatory infiltration, suggesting that PS mitigated HFD-induced hepatic steatosis and inflammation. In addition, Figure 3R–T further demonstrated that PS intervention significantly ameliorated HFD-induced hepatic lesions and steatosis (*P* < 0.05), while the degree of intralobular inflammation showed a downward trend that did not reach statistical significance (*P* > 0.05). These findings are consistent with our clinical trial results, highlighting the efficacy of PS in improving lipid metabolism.

### Phytosterols reduced BSH activity and altered putative BSH-associated microbial taxa

**Figure 4.**
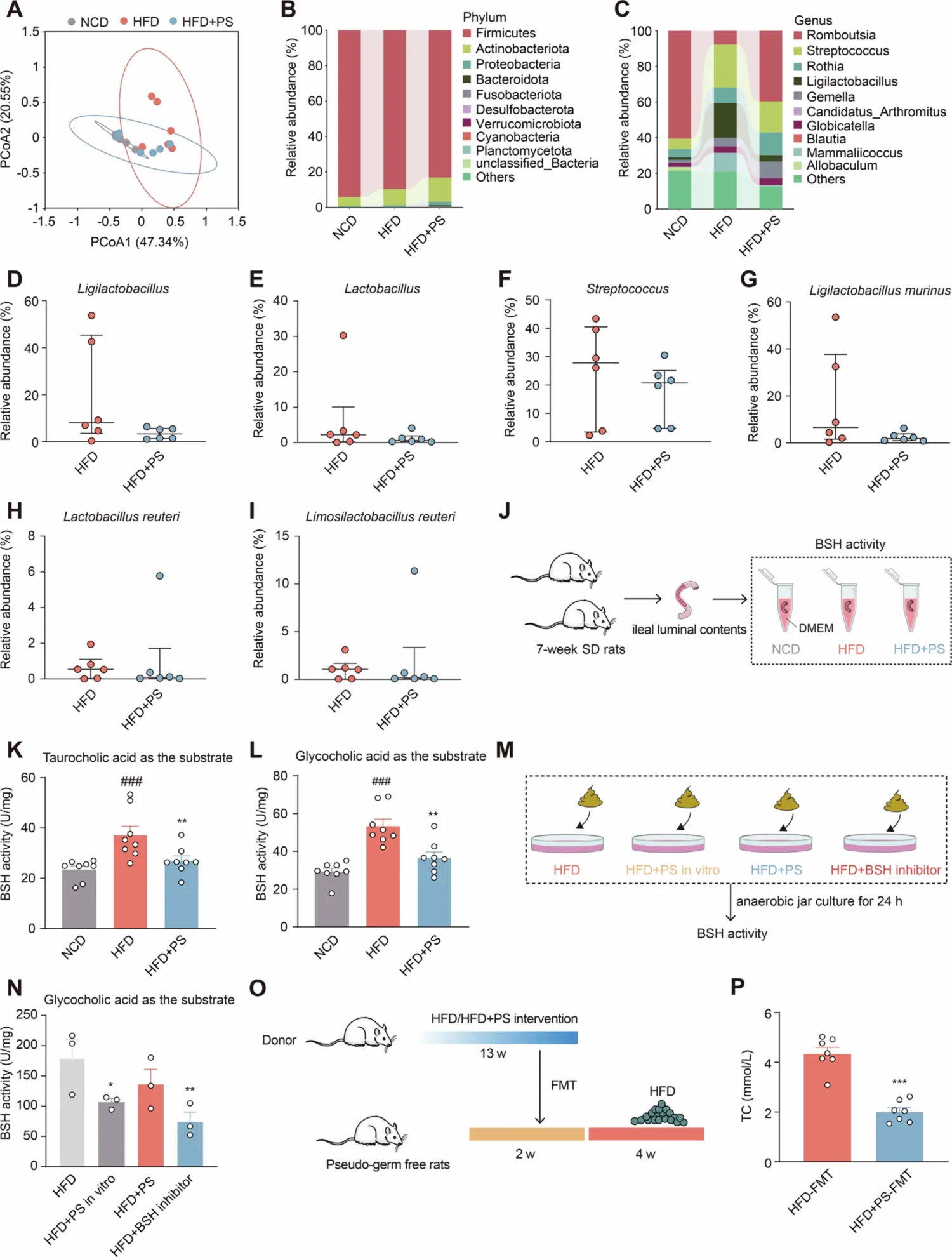
Phytosterols reduce BSH activity and altered putative BSH-associated microbial taxa. (**A**) Principal coordinates analysis (PCoA) of gut microbial communities based on Bray-Curtis dissimilarity (*n* = 6 animals/group). **(B and C)** Gut microbiota composition at the phylum (B) and genus (C) levels. The y-axis indicates the mean relative abundance (%) of taxa within each group. **(D–F)** Relative abundances of *Ligilactobacillus* (D), *Lactobacillus* (E), and *Streptococcus* (F) at the genus level in the HFD and HFD+PS groups. Data are presented as median and interquartile range (IQR). *n* = 6 animals/group. Between-group differences were assessed using the Mann-Whitney U test. **(G–I)** Relative abundances of *Ligilactobacillus murinus* (G), *Lactobacillus reuteri* (H), and *Limosilactobacillus reuteri* (I) at the species level in the HFD and HFD+PS groups. Data are presented as median and interquartile range (IQR). *n* = 6 animals/group. Between-group differences were assessed using the Mann-Whitney U test. (**J**) Schematic of BSH activity measurement in rat ileal luminal contents. (**K and L**) BSH activity measured using taurocholic acid (K) or glycocholic acid (L) as substrates (*n* = 8 animals/group). Data are presented as mean ± SEM. Overall group differences were assessed by one-way ANOVA, with Fisher’s LSD test applied exclusively to pairwise comparisons specified a priori. ^#^ compared to NCD, *P* < 0.05; ^##^ compared to NCD, *P* < 0.01; ^###^ compared to NCD, *P* < 0.001. * compared to HFD, *P* < 0.05; ** compared to HFD, *P* < 0.01; *** compared to HFD, *P* < 0.001. (**M**) Schematic of the in vitro fecal microbiota culture experiment. The experimental groups comprised microbiota from HFD-fed rats (HFD), microbiota from HFD-fed rats treated with PS in vitro (HFD+PS in vitro), microbiota obtained from HFD+PS-fed donor rats (HFD+PS), and microbiota treated with a BSH inhibitor (HFD+BSH inhibitor). All microbiota cultures were maintained under anaerobic conditions using anaerobic culture bags (*n* = 3 independent cultures/group). (**N**) BSH activity measured using glycocholic acid as the substrate (*n* = 3 independent cultures/group). Data are presented as mean ± SEM. Overall group differences were assessed by one-way ANOVA, with Fisher’s LSD test applied exclusively to pairwise comparisons specified a priori. * compared to HFD, *P* < 0.05; ** compared to HFD, *P* < 0.01; *** compared to HFD, *P* < 0.001. (**O**) Schematic of the fecal microbiota transplantation (FMT) experiment in antibiotic-treated recipient rats. HFD-FMT recipients received microbiota from HFD-fed donor rats, whereas HFD+PS-FMT recipients received microbiota from HFD+PS-fed donor rats. (**P**) Serum TC concentration at the end of the FMT experiment (*n* = 7 animals/group). Data are presented as mean ± SEM. Differences between groups were assessed using an unpaired two-sided Student’s *t* test. * compared to HFD-FMT, *P* < 0.05; ** compared to HFD-FMT, *P* < 0.01; *** compared to HFD-FMT, *P* < 0.001.

To further investigate the impact of PS intervention on the gut microbiota of hyperlipidemic rats, we performed 16S rRNA gene sequencing on the ileal contents. While the differences of alpha diversity in all groups did not reach statistical significance, HFD feeding showed a decreasing trend in microbial richness and diversity, whereas PS intervention appeared to reverse this reduction (Figure S4). Figure 4A presents the principle coordinate analysis (PCoA) based on Bray-Curtis distances to evaluate the overall *β*-diversity of gut microbiota among groups. The first two principal coordinates (PCoA1 and PCoA2) explained 47.34% and 20.55% of the total variance, respectively. A clear separation was observed between the NCD and HFD groups, indicating that HFD markedly altered the gut microbial community structure. In contrast, the HFD+PS group clustered more closely with the NCD group, suggesting that PS intervention partially restored the HFD-induced microbial dysbiosis. At the phylum level (Figure 4B), *Firmicutes* dominated the gut microbiota across all groups, while HFD feeding led to a relative increase in *Actinobacteriota*. At the genus level (Figure 4C), HFD feeding markedly altered the microbial profile, characterized by a decreased abundance of *Romboutsia*, and an increased abundance of *Streptococcus* and *Ligilactobacillus*, whereas PS treatment appeared to decrease the relative abundance of *Streptococcus* and *Ligilactobacillus.* Further quantitative comparisons showed that the relative abundance of *Ligilactobacillus*, *Lactobacillus*, and *Streptococcus* at the genus level tended to decrease after PS supplementation compared with the HFD group, although the differences did not reach statistical significance (Figure 4D–F). A similar pattern was observed at the species level, where *Ligilactobacillus murinus*, *Lactobacillus reuteri*, and *Limosilactobacillus reuteri* exhibited downward trends following PS intervention (Figure 4G–I). Although these shifts did not reach statistical significance, this trend hints at a putative suppressive influence of PS on select BSH-producing taxa, which could correlate with downstream remodeling of the bile acid pool. To further examine the effects of PS intervention on the enrichment of putative BSH-producing taxa, we quantified BSH activity in rat ileal luminal contents (Figure 4J). As shown in Figure 4K and L, HFD feeding significantly enhanced BSH activity when either TCA or GCA was used as the substrate. PS supplementation significantly attenuated this elevation, suggesting a suppressive effect on HFD-induced BSH hyperactivity. To determine whether this effect was specifically attributable to PS, we performed ex vivo culture of fecal microbiota derived from HFD- or HFD+PS-fed rats (Figure 4M). Consistently, BSH activity was significantly reduced in the HFD+PS in vitro group compared with HFD alone, and comparable inhibition was observed when a BSH inhibitor was applied (Figure 4N). To determine the role of gut microbiota in the PS-induced improvement of lipid metabolism, the fecal microbiota transplant was conducted (Figure 4O). There was no significant difference in body weight between two groups at the end of the fecal microbiota transplant experiment (Figure S5). However, pseudo-germ-free rats receiving fecal microbiota from HFD+PS donors showed significantly lower serum TC and TG levels compared with those transplanted with HFD-derived microbiota (Figures 4P and S6), suggesting that the cholesterol-lowering effects of PS are partially mediated by the gut microbiota.

### Phytosterols increase THDCA concentrations in liver tissue and ileal contents

**Figure 5.**
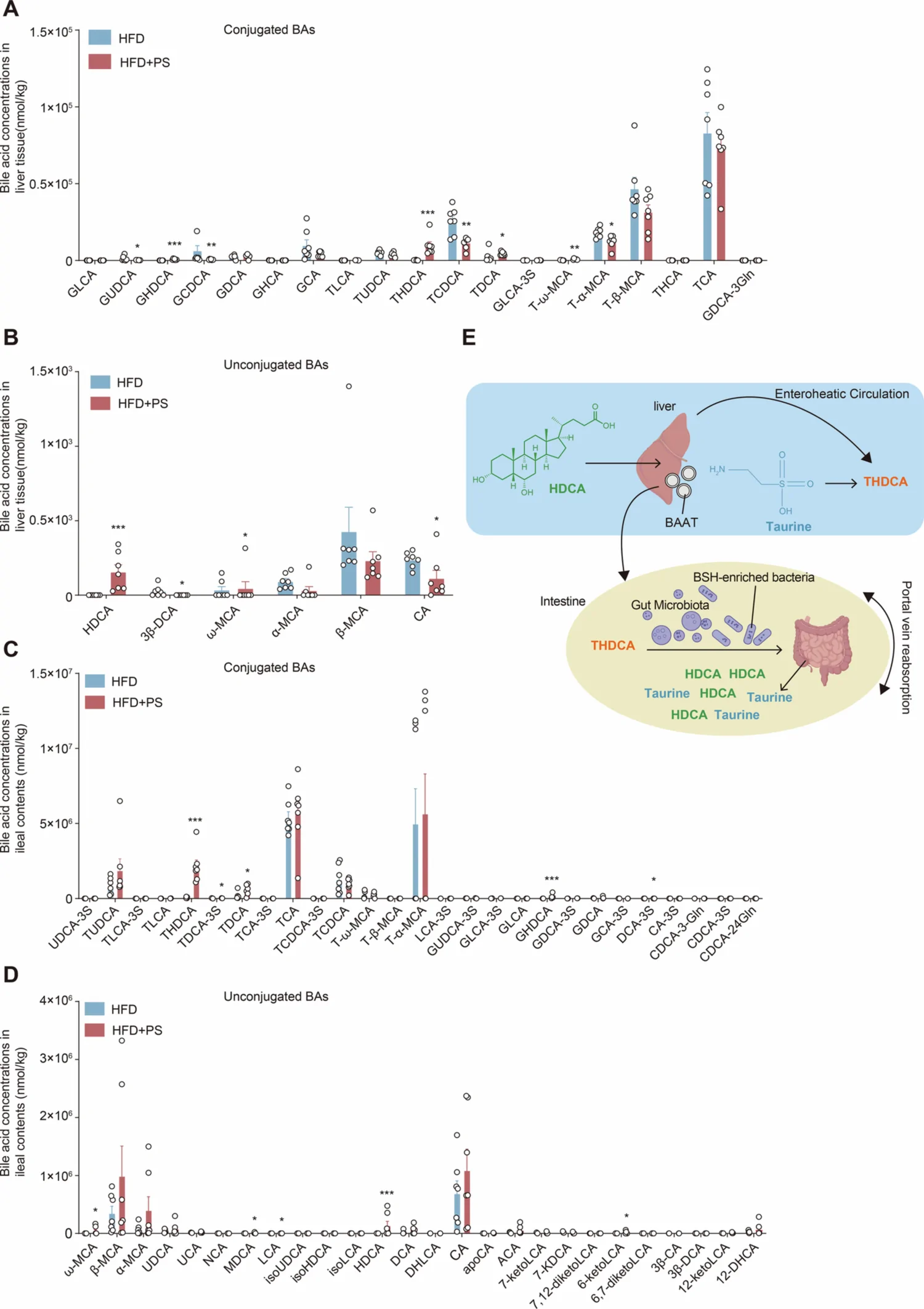
Phytosterols increase THDCA concentrations in liver tissue and ileal contents. **(A and B)** Concentrations of conjugated (A) and unconjugated (B) BAs in liver tissue from the HFD and HFD+PS groups. Data are presented as mean ± SEM (*n* = 7 animals/group). Between-group differences were assessed using the Mann-Whitney U test. * compared to HFD, *P* < 0.05; ** compared to HFD, *P* < 0.01; *** compared to HFD, *P* < 0.001. **(C and D)** Concentrations of conjugated (C) and unconjugated (D) BAs in ileal contents from the HFD and HFD+PS groups. Data are presented as mean ± SEM (*n* = 7 animals/group). Between-group differences were assessed using the Mann-Whitney U test. * compared to HFD, *P* < 0.05; ** compared to HFD, *P* < 0.01; *** compared to HFD, *P* < 0.001. **(E)** Schematic of the proposed enterohepatic interconversion between HDCA and THDCA. In this proposed model, hepatic taurine conjugation of HDCA generates THDCA, which is subsequently secreted into the intestinal lumen via bile. In the gut, microbial BSH may deconjugate THDCA to HDCA, which can then be reabsorbed and returned to the liver through the portal circulation. HDCA, hyodeoxycholic acid; THDCA, taurohyodeoxycholic acid.

To examine the effects of PS supplementation on BA metabolism within the enterohepatic circulation, BA profiles were analyzed in liver tissue and ileal contents (Figure 5A–D). Compared with the HFD group, PS supplementation significantly increased the concentrations of both the taurine-conjugated BA taurohyodeoxycholic acid (THDCA) and its unconjugated counterpart hyodeoxycholic acid (HDCA) in liver tissue and ileal contents (all *P* < 0.001). The parallel increases in these two BAs across the hepatic and intestinal compartments were consistent with altered enterohepatic BA metabolism following PS supplementation. As illustrated in the proposed model in Figure 5E, hepatic taurine conjugation of HDCA generates THDCA, whereas microbial BSH activity in the intestine may facilitate THDCA deconjugation back to HDCA. Together with the reduced ileal luminal BSH activity observed following PS supplementation, the increased THDCA concentrations were consistent with decreased microbial deconjugation of THDCA.

### Phytosterols modulate ileal FXR-FGF15 signaling and hepatic markers consistent with a shift toward the alternative BA synthesis pathway

**Figure 6.**
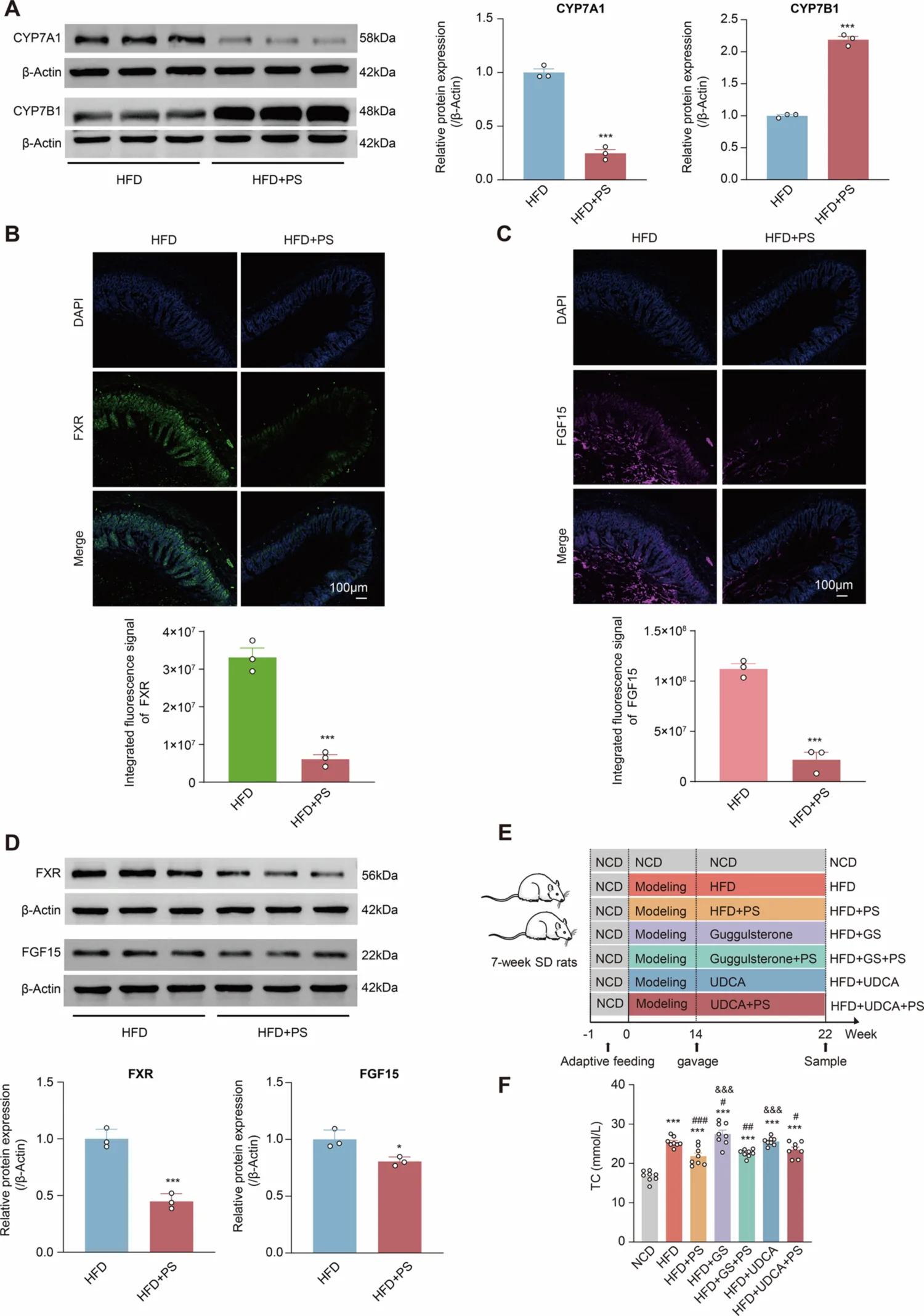
Phytosterols modulate ileal FXR-FGF15 signaling and hepatic markers consistent with a shift toward the alternative BA synthesis pathway. (**A**) Representative western blots and quantification of hepatic CYP7A1 and CYP7B1 protein expression. The decrease in CYP7A1 together with the increase in CYP7B1 was interpreted as a pattern consistent with reduced involvement of the classical pathway and relatively greater involvement of the alternative BA synthesis pathway. Data are presented as mean ± SEM (*n* = 3 biological samples/group). Between-group differences were assessed using an unpaired two-sided Student’s *t* test. * compared to HFD, *P* < 0.05; ** compared to HFD, *P* < 0.01; *** compared to HFD, *P* < 0.001. (**B and C**) Representative immunofluorescence images and quantification of FXR (B) and FGF15 (C) protein expression in ileal tissue, respectively. Data are presented as mean ± SEM (*n* = 3 biological samples/group). Between-group differences were assessed using an unpaired two-sided Student’s *t* test. Scale bar, 100 μm. * compared to HFD, *P* < 0.05; ** compared to HFD, *P* < 0.01; *** compared to HFD, *P* < 0.001. (**D**) Representative western blots and quantification of FXR and FGF15 protein expression in ileal tissue. Data are presented as mean ± SEM (*n* = 3 biological samples/group). Between-group differences were assessed using an unpaired two-sided Student’s *t* test. * compared to HFD, *P* < 0.05; ** compared to HFD, *P* < 0.01; *** compared to HFD, *P* < 0.001. (**E**) Experimental design for the FXR functional study. Rats were assigned to the NCD, HFD, HFD+PS (500 mg/kg/day PS), HFD+GS (50 mg/kg/day Z-guggulsterone), HFD+GS+PS, HFD+UDCA (50 mg/kg/day ursodeoxycholic acid), or HFD+UDCA+PS group (*n* = 8 animals/group). (**F**) Serum TC concentration at the end of the experiment (*n* = 8 animals/group). Data are presented as mean ± SEM. Overall group differences were assessed by one-way ANOVA, with Fisher’s LSD test applied exclusively to pairwise comparisons specified a priori. * compared to NCD, *P* < 0.05; ** compared to NCD, *P* < 0.01; *** compared to NCD, *P* < 0.001. ^#^ compared to HFD, *P* < 0.05; ^##^ compared to HFD, *P* < 0.01; ^###^ compared to HFD, *P* < 0.001. ^&^ compared to HFD+PS, *P* < 0.05; ^&&^ compared to HFD+PS, *P* < 0.01; ^&&&^ compared to HFD+PS, *P* < 0.001.

To further investigate downstream signaling associated with PS-induced BA remodeling, we examined ileal FXR-FGF15 signaling and hepatic proteins related to BA synthesis. As shown in Figures 6A and S7, PS supplementation significantly decreased hepatic CYP7A1 protein expression while increasing CYP7B1 expression compared with the HFD group, a pattern consistent with reduced involvement of the classical pathway and relatively greater involvement of the alternative bile acid synthesis pathway. Immunofluorescence analysis revealed that PS significantly suppressed the ileal tissue expression of FXR and FGF15 (Figure 6B and C). The fluorescence intensities of both FXR and FGF15 were significantly reduced in the PS-treated group relative to HFD group (*P* < 0.05). Western blot analysis showed concordant reductions in ileal FXR and FGF15 protein expression (Figures 6D and S8). To further assess the potential involvement of FXR-related signaling in PS-associated cholesterol lowering, pharmacological intervention was performed using Z-Guggulsterone (a non-selective FXR antagonist) and ursodeoxycholic acid (UDCA, a context-dependent FXR agonist^22^ (Figure 6E). Consistent with our primary findings, PS supplementation significantly reduced serum TC in HFD-fed rats compared with HFD group (Figure 6F). Serum TC did not differ significantly between the HFD+PS and HFD+GS+PS groups (*P* > 0.05). Similarly, serum TC did not differ significantly between the HFD+PS and HFD+UDCA+PS groups (*P* > 0.05). Z-guggulsterone alone did not reproduce the TC-lowering effect observed with PS supplementation. Collectively, these pharmacological findings were compatible with a potential involvement of intestinal FXR-related signaling in the cholesterol-lowering effects associated with PS supplementation.

### THDCA modulates ileal FXR-FGF15 signaling and hepatic markers consistent with a shift toward the alternative BA synthesis pathway in HFD-fed rats

**Figure 7.**
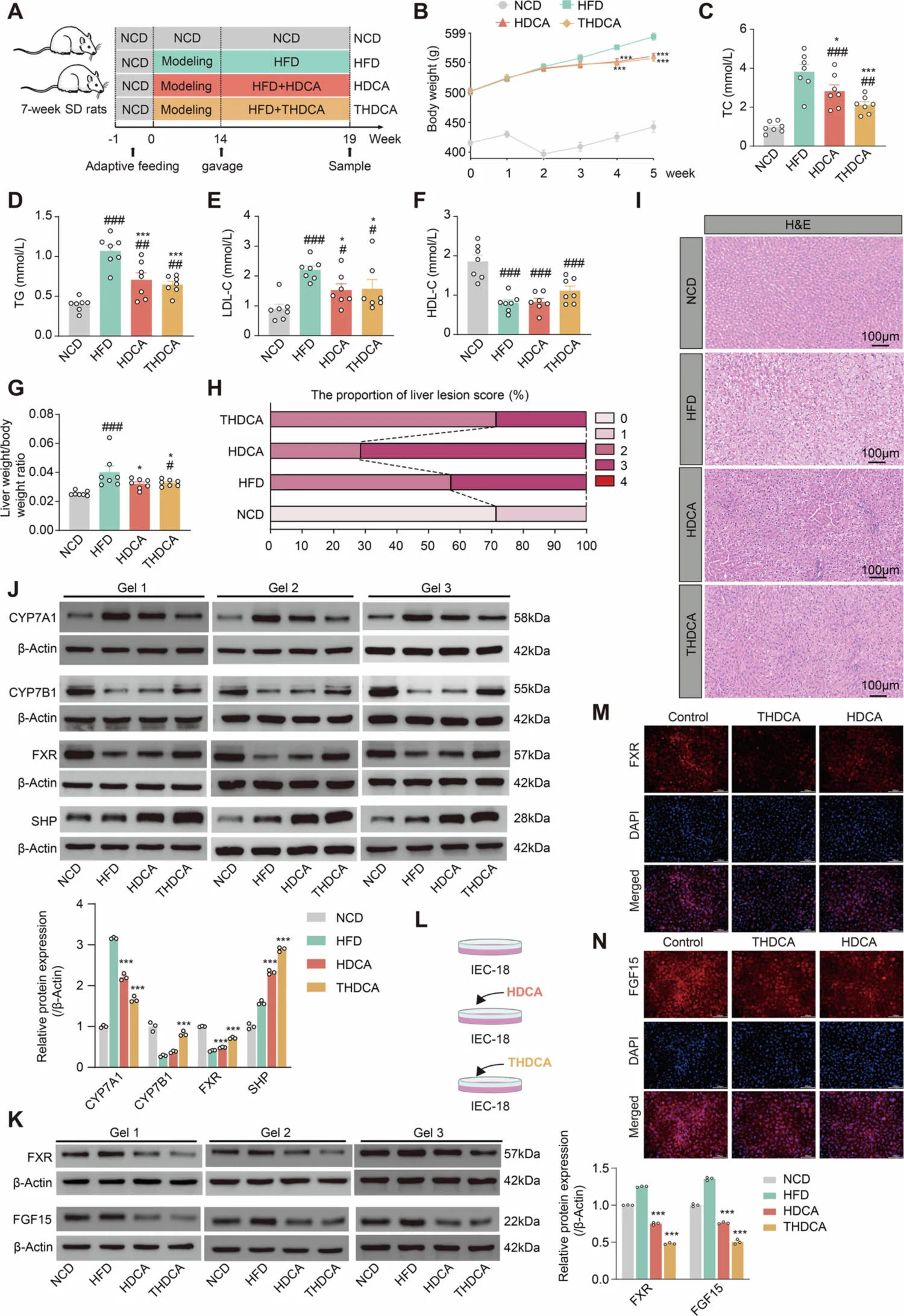
THDCA modulates ileal FXR-FGF15 signaling and hepatic markers consistent with a shift toward the alternative BA synthesis pathway in HFD-fed rats. (**A**) Experimental groups: NCD, HFD, HFD+HDCA (75 mg/kg/day HDCA), and HFD+THDCA (75 mg/kg/day THDCA) (*n* = 7 animals/group). (**B**) Changes in body weight during the 5-week intervention. Data are presented as mean ± SEM. At each point, overall group differences were assessed by one-way ANOVA, with Fisher’s LSD test applied exclusively to pairwise comparisons specified a priori. * compared to HFD, *P* < 0.05; ** compared to HFD, *P* < 0.01; *** compared to HFD, *P* < 0.001. (**C–F**) Serum concentrations of TC, TG, LDL-C, and HDL-C after 5 weeks of intervention, respectively (*n* = 7 animals/group). Data are presented as mean ± SEM. Overall group differences were assessed by one-way ANOVA, with Fisher’s LSD test applied exclusively to pairwise comparisons specified a priori. ^#^ compared to NCD, *P* < 0.05; ^##^ compared to NCD, *P* < 0.01; ^###^ compared to NCD, *P* < 0.001. * compared to HFD, *P* < 0.05; ** compared to HFD, *P* < 0.01; *** compared to HFD, *P* < 0.001. (**G**) Liver-to-body weight ratio (*n* = 7 animals/group). Data are presented as mean ± SEM. Overall group differences were assessed by one-way ANOVA, with Fisher’s LSD test applied exclusively to pairwise comparisons specified a priori. ^#^ compared to NCD, *P* < 0.05; ^##^ compared to NCD, *P* < 0.01; ^###^ compared to NCD, *P* < 0.001. * compared to HFD, *P* < 0.05; ** compared to HFD, *P* < 0.01; *** compared to HFD, *P* < 0.001. (**H**) Representative H&E-stained liver sections. Scale bar, 100 μm. (**I**) Distribution of liver lesion scores in each group (*n* = 7 animals/group). Hepatic lesions were scored on a 0-4 scale, with higher scores indicating greater pathological severity. Overall differences among groups were assessed using the Kruskal-Wallis H test, followed by Dunn's test for pairwise comparisons. ^#^ compared to NCD, *P* < 0.05; ^##^ compared to NCD, *P* < 0.01; ^###^ compared to NCD, *P* < 0.001. * compared to HFD, *P* < 0.05; ** compared to HFD, *P* < 0.01; *** compared to HFD, *P* < 0.001. (**J**) Representative western blots and quantification of hepatic CYP7A1, CYP7B1, FXR, and SHP protein expression. The decrease in CYP7A1 together with the increase in CYP7B1 was interpreted as a pattern consistent with reduced involvement of the classical pathway and relatively greater involvement of the alternative BA synthesis pathway. Data are presented as mean ± SEM (*n* = 3 biological samples/group). Overall group differences were assessed by one-way ANOVA, with Fisher’s LSD test applied exclusively to pairwise comparisons specified a priori. * compared to HFD, *P* < 0.05; ** compared to HFD, *P* < 0.01; ***compared to HFD, *P* < 0.001. Note that all groups (HFD, HFD+HDCA and HFD+THDCA) differed significantly from NCD (*P* < 0.05, not shown). (**K**) Representative western blots and quantification of FXR and FGF15 protein expression in ileal tissue. Data are presented as mean ± SEM (*n* = 3 biological samples/group). Overall group differences were assessed by one-way ANOVA, with Fisher’s LSD test applied exclusively to pairwise comparisons specified a priori. * compared to HFD, *P* < 0.05; ** compared to HFD, *P* < 0.01; ***compared to HFD, *P* < 0.001. Note that all groups (HFD, HFD+HDCA and HFD+THDCA) differed significantly from NCD (*P* < 0.05, not shown). (**L**) Schematic of the in vitro intervention experiment using rat ileal cells. Experiments were performed with three independent replicates per group. (**M and N**) Representative immunofluorescence images of FXR (M) and FGF15 (*N*) protein expression in rat ileal cells, respectively. Scale bar, 100 μm.

To investigate the effects of HDCA and its taurine-conjugated derivative THDCA on ileal FXR-FGF15 signaling, rats received HDCA or THDCA supplementation alongside an HFD (Figure 7A). Compared with the HFD group, body weight was significantly lower in the HFD+HDCA and HFD+THDCA groups after 4 weeks of intervention (Figure 7B). THDCA and HDCA supplementation significantly reduced serum TC, TG, and LDL-C compared with the HFD group (Figure 7C–F). HFD feeding also increased the liver weight-to-body weight ratio, whereas HDCA or THDCA treatment reduced this effect (Figure 7G). Histological assessment using H&E staining revealed substantial hepatic steatosis and ballooning degeneration in the HFD group, whereas both HDCA and THDCA treatment attenuated these histopathological alterations. Consistent with the histological findings, liver lesion scores were lower following HDCA or THDCA supplementation than in the HFD group (Figure 7H and I). Western blot analysis showed that HDCA and THDCA treatment decreased hepatic CYP7A1 protein expression while increasing CYP7B1 expression, a pattern consistent with reduced involvement of the classical pathway and relatively greater involvement of the alternative BA synthesis pathway, similar to that observed following PS supplementation. HDCA and THDCA treatment also increased hepatic FXR and SHP protein expression (Figures 7J and S9). In contrast, HDCA or THDCA treatment significantly reduced ileal FXR and FGF15 protein expression compared with the HFD group (Figures 7K and S10). To further assess whether HDCA and its taurine-conjugated counterpart THDCA could directly modulate FXR-FGF15 signaling in intestinal epithelial cells, IEC-18 cells were treated with HDCA or THDCA (Figure 7L). Immunofluorescence analysis showed significantly reduced FXR and FGF15 fluorescence intensities in HDCA- and THDCA-treated IEC-18 cells compared with the control group (Figure 7M and N), suggesting that these two BAs may suppress intestinal FXR-FGF15 signaling at the cellular level. Together, these findings indicate that HDCA and THDCA ameliorated HFD-associated dyslipidemia and hepatic pathological changes while suppressing ileal FXR-FGF15 signaling and inducing hepatic CYP7A1/CYP7B1 expression patterns consistent with a relative shift from the classical toward the alternative BA synthesis pathway.

### Exploratory human relevance of phytosterol-associated serum BA remodeling

**Figure 8.**
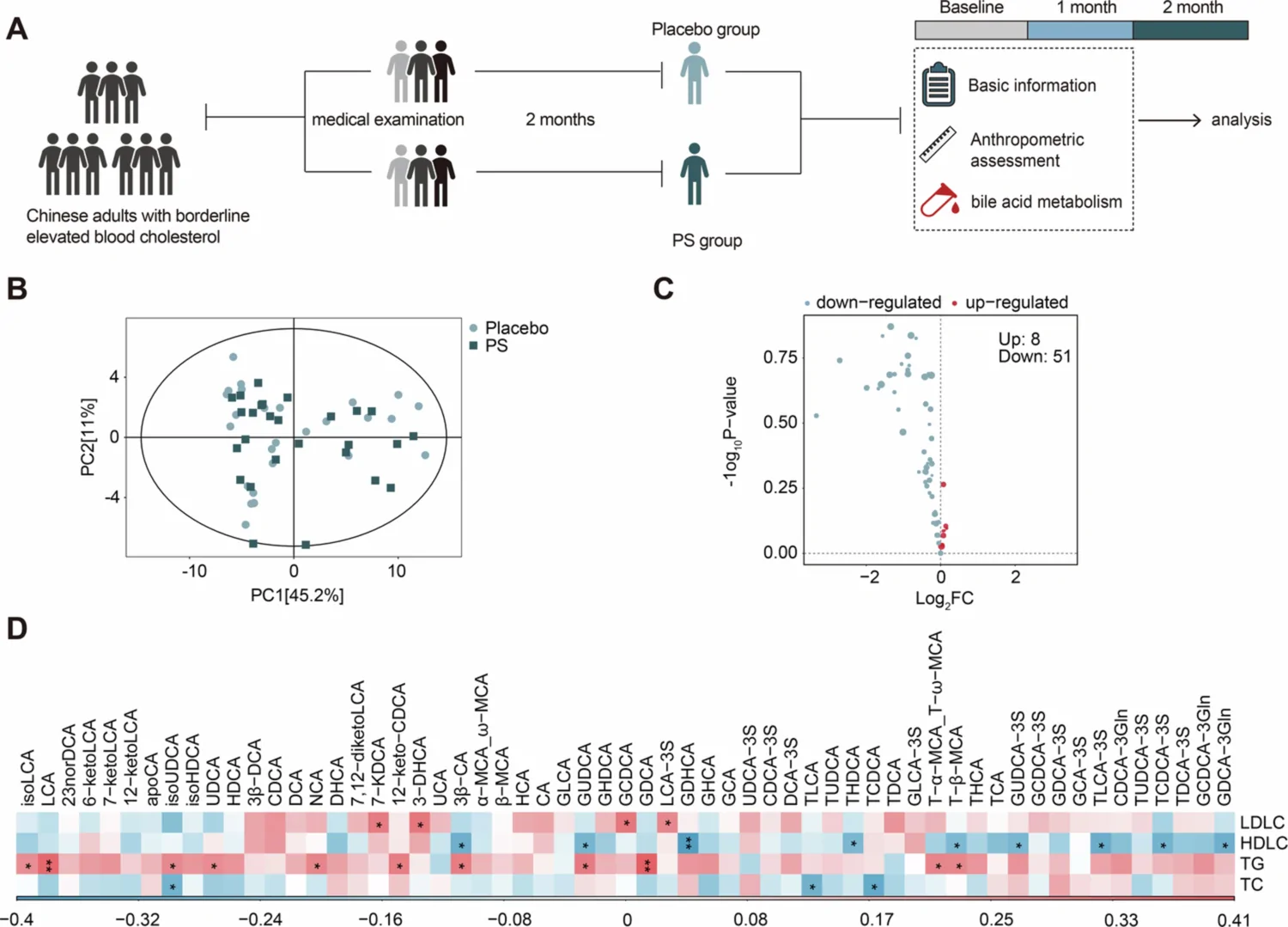
Exploratory human relevance of phytosterol-associated serum BA remodeling. (**A**) Schematic of the randomized, placebo-controlled intervention trial. Participants in the PS group received phytosterol-containing capsules, whereas those in the placebo group received wheat starch-containing capsules (*n* = 27 participants/group). Fecal samples were not collected in this trial; therefore, gut microbiota composition and fecal BSH activity were not assessed, and the human analysis was restricted to circulating BA profiles and lipid-related outcomes. (**B**) Principal component analysis (PCA) score plot of serum BA profiles in the PS and placebo groups after 1 month of intervention. (**C**) Volcano plot illustrating exploratory differences in serum BA profiles between the PS and placebo groups after 1 month of intervention. (**D**) Spearman correlation heatmap showing associations between serum lipid parameters and serum BA concentrations after 1 month of intervention across all participants. *P < 0.05; **P < 0.01.

**Figure 9.**
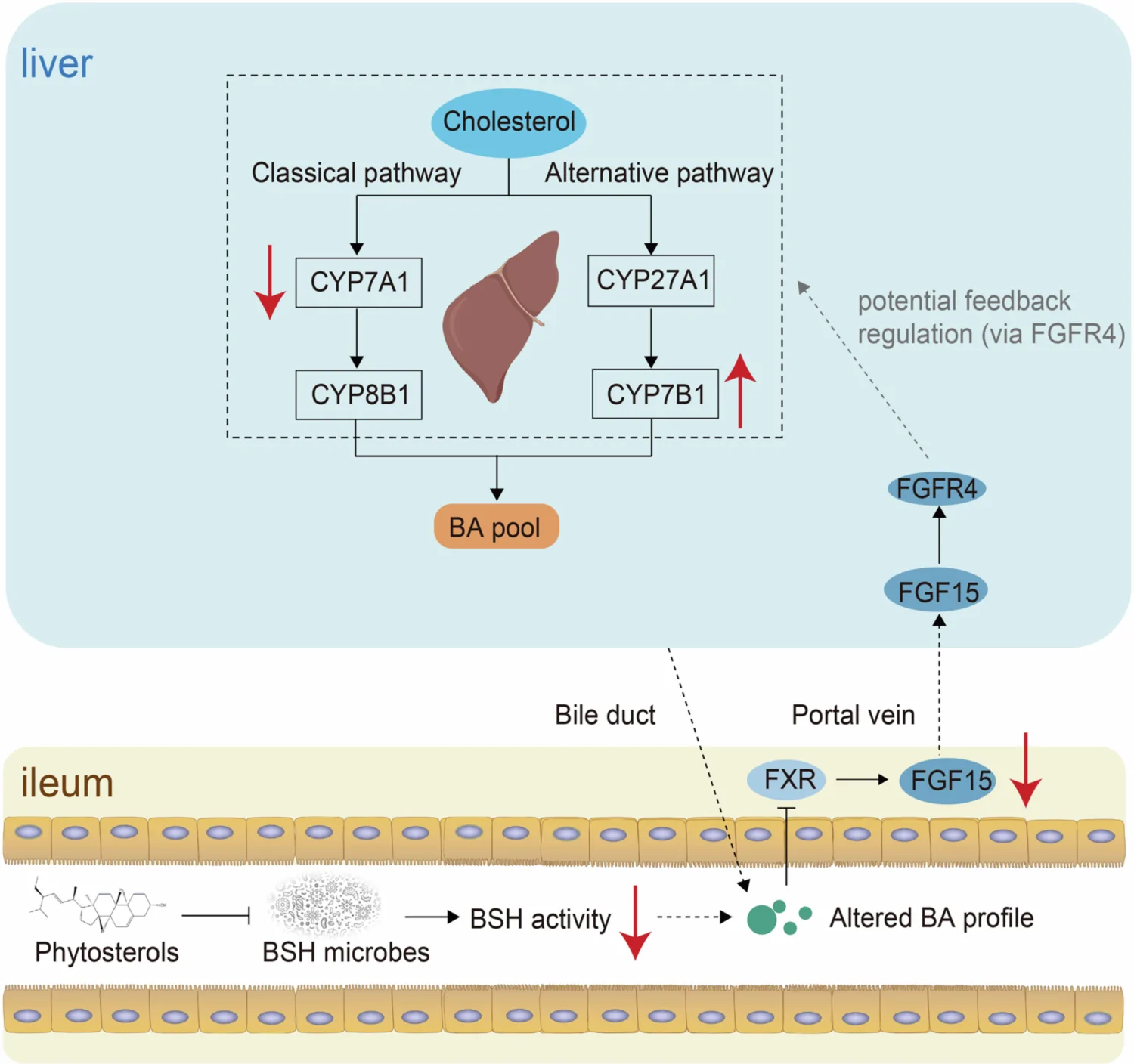
Proposed working model of phytosterol-mediated improvement of dyslipidemia involving gut microbiota-associated BSH activity, BA remodeling, and enterohepatic FXR signaling.

To explore the clinical relevance of the BA alterations observed in animal experiments, we retrospectively analyzed banked serum samples from a previously reported randomized, placebo-controlled trial.^20^ We focused our analysis on participants with borderline hypercholesterolemia who received either PS (2 g/day) or placebo (Figure 8A). PS supplementation reduced serum TC concentrations compared with placebo, consistent with the established lipid-lowering effect of PS in humans. To further examine BA responses, serum BA profiles were characterized by UHPLC-MRM-MS after the 1-month intervention. Although no clear overall separation in serum BA profiles was observed between the PS and placebo groups (Figure 8B and C, Table S11), exploratory correlation analyzes identified associations between several serum BA concentrations and lipid parameters after the intervention (Figure 8D). These findings suggest a potential relationship between circulating BA patterns and lipid phenotypes, but do not establish that BA changes mediated the lipid-lowering effect of PS. Together with the preceding dietary intervention and experimental studies, these exploratory human data provide translational support for an association between PS exposure, lipid improvement, and circulating BA remodeling. Based on the collective human and experimental findings, we proposed an integrated working model in which gut microbiota-associated BSH activity, BA remodeling, and enterohepatic FXR signaling may contribute to the lipid-modulating effects of PS (Figure 9).

## Discussion

This study adopts a multi-level translational framework spanning population epidemiology, dietary intervention trials and preclinical mechanistic exploration, to characterize lipid-modulating effects of dietary PS and a complementary underlying metabolic pathway. Specifically, our translational workflow originates from real-world population observations and pragmatic food-based interventions, where PS were naturally incorporated into daily edible oils rather than administered as isolated purified nutrients, better reflecting habitual dietary patterns. By synthesizing data from three independent human studies, we observe consistent lipid-lowering trends associated with PS exposure across distinct research designs: cross-sectional analysis of habitual dietary PS intake, an RCT using PS-fortified corn and wheat germ blended oil, and a further trial supplying purified PS supplements. These consistent clinical associations guided subsequent animal experiments, which explored gut microbiota-BA interactions as potential mechanistic mediators. Preclinical assays identified a putative gut microbiota-BA-FXR signaling cascade that may contribute to the lipid-regulating properties of dietary PS.

While the classical mechanism of PS involves the competitive inhibition of intestinal cholesterol absorption, our preclinical data suggest that PS may also modulate gut microbial composition and function. This mechanistic framework offers a tentative explanation for well-documented interindividual variation in PS responsiveness,^23^ implying that baseline gut microbial profiles may represent one factor contributing to interindividual variation in responses to PS supplementation. In this context, our observations add a microbiome-related layer to established gene-diet interaction models, suggesting gut microbiota as one putative intermediate influencing metabolic responses to dietary PS.^24^ Importantly, the primary delivery vehicle in human trials was PS-enriched corn-wheat germ blended oil, whose fatty acid composition differs substantially from the low-PS control peanut oil. The fortified oil contained approximately 1.86-fold higher linoleic acid and 47% lower oleic acid relative to the control oil. Differences in fatty acid profiles are known to alter gut microbiota, BA metabolism and systemic lipid homeostasis,^25^ and such confounding lipid effects cannot be fully ruled out when interpreting human cohort results. In animal models, we identified a potential microbial biomarker linked to altered BSH activity upon PS exposure. Animal-derived data indicate that PS supplementation correlates with the reduced relative abundance of putative BSH-associated taxa (e.g., *Ligilactobacillus*, *Lactobacillus*, *Streptococcus*), accompanied by elevated conjugated BAs (such as THDCA). These conjugated BAs may act as mild intestinal FXR antagonists, capable of dampening the FXR-FGF15 signaling cascade and relieving feedback suppression of hepatic BA synthesis. This aligns with prior work reporting that HDCA, a secondary BA whose conjugated derivatives include THDCA,^26^ can inhibit intestinal FXR signaling, upregulate hepatic CYP7B1 and facilitate cholesterol catabolism via the alternative BA synthesis pathway.^27^ HDCA concentrations are frequently depleted in metabolic diseases including non-alcoholic fatty liver disease and drug-induced liver injury, and exogenous HDCA supplementation can alleviate hepatic steatosis and oxidative stress in preclinical systems.^28^ To separate PS-specific effects from confounding fatty acid signals and explore potential causal pathways, follow-up animal studies utilized purified PS as the sole intervention under strictly controlled dietary conditions. This experimental design allowed targeted assessment of PS-related changes to the gut microbiota-BA-FXR axis. Integrating preclinical mechanistic findings with human RCT associations provides preliminary translational insights, which may inform food-based nutritional strategies for managing dyslipidemia.

Gut microbial BA biotransformation represents an important regulatory node for systemic metabolism.^29^ In our animal experiments, PS intervention lowered ileal BSH enzymatic activity, a change correlated with reduced relative abundance of *Ligilactobacillus*, *Lactobacillus*, and *Streptococcus*. This aligns with previous rodent research reporting suppressed BSH-producing *Lactobacillus* following PS supplementation under high-fat feeding, alongside altered cholesterol metabolism.^30^ BSH is a key enzyme catalyzing BA deconjugation; suppressed BSH activity tends to preserve pools of conjugated bile acids.^31^ Unlike prior work focusing broadly on overall microbial diversity,^32^ ^,^ ^33^ our analysis highlights a shift in a specific microbial functional trait related to BSH activity. Although dietary fatty acids can remodel gut microbial communities and BA pools,^34^ ^,^ ^35^ trials with purified PS in controlled animal diets consistently reduced intestinal BSH activity. These preclinical results imply that PS may modulate microbial BSH function even under conditions in which fatty acid composition was controlled. Prior pharmacological research has shown synthetic BSH inhibitors can improve metabolic phenotypes in animal models^36^; by comparison, our rodent data indicate dietary PS may modestly reduce intestinal microbial BSH activity, acting as a distinct gut modulator rather than a targeted pharmacological agent. Notably, robust clinical evidence verifying direct BSH-suppressive effects of PS in humans remains absent. Collectively, these animal-derived observations suggest the metabolic benefits of PS are not limited to luminal cholesterol binding and may also relate to modified gut microbial enzymatic function.

Another key preclinical observation involves accumulated conjugated BAs, particularly THDCA, and their capacity to dampen intestinal FXR signaling. While intestinal FXR activation yields favorable metabolic outcomes in certain metabolic contexts,^37^ our rodent data support the hypothesis that partial intestinal FXR inhibition is associated with remodeling of hepatic BA synthesis pathways. PS-induced THDCA accumulation attenuates downstream hepatic FGF15 signaling, leading to elevated CYP7B1 and suppressed CYP7A1 expression. This suggests a multi-layered feedback network in which PS-associated suppression of ileal FXR-FGF15 signaling is accompanied by hepatic CYP7A1/CYP7B1 expression patterns consistent with a relative shift from the classical toward the alternative BA synthesis pathway. This proposed pathway offers a tentative molecular explanation for PS lipid-lowering effects even with minimal systemic absorption of PS.^38^ CYP7B1 plays an important role in regulating oxysterol homeostasis; it converts 27-hydroxycholesterol into BA precursors and prevents buildup of bioactive oxysterols linked to lipotoxicity and disrupted lipid sensing.^39^ Consistent with this, HDCA specifically upregulates hepatic CYP7B1, supporting the contribution of this BA species to the alternative cholesterol catabolic pathway.^27^ Intriguingly, upregulation of the alternative CYP7B1 pathway coincides with suppressed expression of the classical pathway enzyme CYP7A1. Depleted BA pools would typically trigger compensatory CYP7A1 upregulation to maintain BA homeostasis.^40^ The observed CYP7A1 suppression in our rodent model raises the possibility that THDCA and other PS-modulated bile acids may partially repress the classical BA synthesis pathway or reflect a metabolic shift prioritizing the alternative cholesterol disposal route. HDCA has also been shown to inhibit CYP7A1 via a gut microbiota-PPARα-dependent mechanism, further illustrating complex layered regulation of BA synthesis.^27^ This shift toward alternative BA synthesis likely remodels overall BA pool composition, which may broadly influence intestinal lipid handling. Recent work demonstrates that BA pool size and composition selectively regulate dietary fatty acids absorption and secretion of gut hormones like GLP-1.^41^ Accordingly, redirecting cholesterol flux toward CYP7B1-mediated alternative synthesis and limiting CYP7A1-driven BA synthesis may contribute to altered hepatic cholesterol disposal while shaping BA pool composition.

Beyond describing a previously underappreciated gut-liver signaling cascade, our data add complementary context to well-studied PS dietary interventions. While cholesterol-lowering effects of PS have been documented for decades,^42^ the gut microbiota-BA-FXR pathway characterized here complements the classical cholesterol absorption inhibition model and provides an additional framework to interpret PS-related metabolic changes. First, our preclinical mechanistic data expand upon the canonical luminal absorption-blocking model. Rodent experiments indicate PS may modulate the microbial BSH-bile acid-FXR cascade under controlled dietary conditions, with effects extending beyond intestinal cholesterol binding to indirectly alter hepatic cholesterol turnover through gut microbiota-associated BA signaling. Second, our findings put forward a tentative molecular hypothesis explaining heterogeneous individual responses to PS supplementation. We propose that baseline gut communities enriched with putative BSH-associated taxa may represent one factor contributing to variable lipid-lowering responses across participants. Notably, formal responder/non-responder subgroup stratification and statistical testing were not performed within our human trials; this microbial link remains a preclinical-derived conjecture and requires dedicated stratified trials for validation. From a speculative mechanistic perspective, the gut microbiome may act as one candidate factor shaping responses to precision nutritional PS interventions, with potential implications for personalized lipid management research. Finally, our animal data indicate a mild modulatory interaction between PS and gut bacterial BSH enzymes, which may guide future research into nutritional strategies targeting BA turnover, without framing PS as a clinically validated BSH inhibitor for metabolic treatment. Unlike synthetic BSH inhibitors with potential off-target pharmacological risks, dietary PS interact with gut bacteria via endogenous metabolic pathways to shift BA pools, which may inspire mild food-based approaches for mitigating metabolic abnormalities in preclinical settings.

The translational value of this work is supported by its bidirectional bench-to-bedside research design. Translating mechanistic preclinical findings into practical dietary guidance remains a major challenge in nutritional science; however, consistent associations observed across our human RCTs suggest that incorporating PS into staple foods such as cooking oil represents a promising public health approach worthy of further large-scale verification. Compared with pharmaceutical lipid-lowering agents or isolated oral supplement tablets, food-based PS interventions may offer a practical approach for incorporating phytosterols into habitual diets and warrant further evaluation in dyslipidemia management. Clinically, characterizing these non-absorptive gut microbial pathways may open new research avenues for integrated lipid management. PS-fortified foods carry a favorable safety profile and could serve as a supportive lifestyle intervention, particularly for individuals with borderline dyslipidemia where pharmaceutical therapy is not yet indicated.

We acknowledge several key limitations of the present study. First, our mechanistic validation mainly relied on animal models. Although pharmacological inhibitors were applied to characterize key regulatory pathways, these chemical interventions harbor potential off-target effects and lack absolute specificity. The absence of genetic knockout experiments further limits our ability to precisely define the indispensable role of individual nodes within the gut-liver BA regulatory axis. Second, translational human evidence remains incomplete. Human analyzes were limited to serum BA profiles and fecal microbiota sequencing, lacking direct measurements of intestinal BA concentrations, fecal BSH activity or intestinal FXR/FGF19 signaling. All observed microbial and metabolic shifts in human participants are correlational and cannot confirm causal mechanistic pathways. Third, microbiota profiling was based on 16S rRNA gene sequencing rather than shotgun metagenomics. Full-length 16S rRNA sequencing was applied to the animal samples, providing improved taxonomic resolution and enabling species-level annotations. Nevertheless, 16S-based taxonomic profiling does not directly establish the presence or abundance of BSH-encoding genes or microbial BSH functional capacity. Future shotgun metagenomic sequencing will therefore be required to validate taxonomic assignments and directly characterize BSH-related functional changes. Fourth, notable interspecies differences exist in BA regulatory signaling; rodent FGF15 and human FGF19 exhibit divergent biological functions, limiting direct extrapolation of rodent mechanistic results to human physiology. Furthermore, different edible oil vehicles were used across human intervention arms, introducing potential confounding from distinct fatty acid profiles. Although purified PS animal experiments isolated PS-specific bioactivity, residual background dietary interference cannot be fully eliminated. Finally, multiplicity correction was not applied to the exploratory metabolomic and microbiome analyzes, which may increase the risk of false-positive findings; these results should therefore be interpreted as exploratory and require independent validation.

## Conclusion

In conclusion, the present study proposes a working mechanistic framework in which dietary phytosterols may partially ameliorate dyslipidemia through processes involving modulation of gut microbial BSH activity, bile acid remodeling, and enterohepatic FXR signaling. Human observational and intervention data provide exploratory evidence linking phytosterol exposure with favorable lipid profiles and circulating bile acid changes, whereas complementary animal and cellular experiments support THDCA-associated modulation of FXR-FGF15 signaling as one plausible contributing mechanism. These experimental findings were accompanied by decreased hepatic CYP7A1 and increased CYP7B1 expression, a pattern consistent with a relative shift from the classical toward the alternative bile acid synthesis pathway. Taken together, the human and preclinical findings suggest that gut microbiota-bile acid crosstalk may represent one pathway contributing to the lipid-modulatory effects of phytosterols. These findings provide a rationale for future dedicated clinical studies to further investigate microbiota-bile acid signaling in the nutritional management of dyslipidemia.

## Supplementary Material

supplemantary appendix.docx supplemantary appendix.docx

## Data Availability

The raw 16S rRNA gene sequencing data generated in this study have been deposited at the National Center for Biotechnology Information (NCBI), accessible via https://www.ncbi.nlm.nih.gov/sra/PRJNA1401276 and https://www.ncbi.nlm.nih.gov/sra/PRJNA1404683. The metabolomics data have been deposited in the MetaboLights repository, accessible via https://www.ebi.ac.uk/metabolights/MTBLS13654, https://www.ebi.ac.uk/metabolights/MTBLS13656, https://www.ebi.ac.uk/metabolights/MTBLS13655 and https://www.ebi.ac.uk/metabolights/MTBLS13652. The study protocol and the datasets created and/or examined during this study will be available on reasonable requests.
