# Supplementary material for: Phytosterol-induced modulation of gut microbial bile salt hydrolases ameliorates hyperlipidemia via taurohyodeoxycholic acid-mediated FXR antagonism
Source: Gut Microbes. 2026 Sep 17;18(1):2731687. doi: 10.1080/19490976.2026.2731687 (PMC13596926; doi:10.1080/19490976.2026.2731687)
Supplement: Supplementary file 1 [file KGMI_A_2731687_SM3135.docx]

**Supplementary appendix**

**Supplement to:**

**Phytosterol-induced modulation of gut microbial bile salt hydrolases ameliorates hyperlipidemia via taurohyodeoxycholic acid-mediated FXR antagonism**

Jiayue Xia^1,2#^, Da Pan^1,2#^, Tianyu Wu^1,2#^, Zhiyuan Feng^1,2#^, Yanyan Tian^1,2^, Zhi He^3^, Zhenzhen Zhang^1,2^, Yingqi Shao^1,2^, Junhui Yu^1,2^, Wei Yan^1,2^, Jiongnan Wang^1,2^, Shiyu Yin^1,2^, Xingyi Jin^1,2^, Yilu Chen^1,2^, Yuanyuan Wang^1,2^ and Guiju Sun^1,2*^

^1^ Key Laboratory of Environmental Medicine and Engineering of Ministry of Education, School of Public Health, Southeast University, Nanjing 210009, P.R. China

^2^ Department of Nutrition and Food Hygiene, School of Public Health, Southeast University, Nanjing 210009, P.R. China

^3^ Zhongda Hospital Affiliated to Southeast University, Nanjing 210009, China

^#^ These authors contributed equally to this work and share co-first authorship.

**Table S1**. The Phytosterol content of selected food from the Sixth Edition of the Chinese Food Composition Table (mg/100 g of edible portion)

| **Number** | **Food name** | **Cereal sterol (β-sitosterol)** | **Camellia sterol** | **Sterol** | **Cereal steranol** | **Rapesanol** | **Rapeseed steranol** | **Total** |
| --- | --- | --- | --- | --- | --- | --- | --- | --- |
|  | **Cereals and Their Products** |  |  |  |  |  |  |  |
| Ps001 | whole-wheat flour | 48.08 | 13.46 | 1.81 | 14.66 | 7.49 | Tr | 85.49 |
| Ps002 | standard wheat flour | 38.00 | 9.55 | 1.49 | 10.33 | 5.50 | Tr | 64.07 |
| Ps003 | bread flour / high-gluten flour | 29.36 | 6.24 | 0.96 | 10.85 | 4.80 | Tr | 52.21 |
| Ps004 | dumpling flour | 28.90 | 5.45 | 1.65 | 8.60 | 4.22 | Tr | 48.75 |
| Ps005 | Xiaozhan rice | 9.32 | 2.49 | 2.37 | 1.60 | Tr | Tr | 15.78 |
| Ps006 | glutinous rice | 5.21 | 2.01 | 1.35 | 3.94 | 1.40 | Tr | 13.90 |
| Ps007 | fragrant rice | 5.47 | 1.82 | 2.35 | 1.84 | 0.69 | Tr | 12.16 |
| Ps008 | milled rice / white rice | 6.65 | 2.52 | 1.32 | 1.63 | 0.32 | Tr | 12.28 |
|  | **Legumes and Their Products** |  |  |  |  |  |  |  |
| Ps009 | soybean | 64.98 | 25.55 | 17.18 | 5.15 | 1.11 | Tr | 114.54 |
| Ps0010 | green soybean | 56.36 | 14.64 | 9.48 | 4.61 | 1.02 | Tr | 86.12 |
| Ps0011 | black soybean | 53.40 | 13.06 | 10.34 | 6.00 | 1.04 | Tr | 83.84 |
| Ps0012 | mung bean | 38.92 | 7.03 | 2.55 | 10.93 | 4.64 | Tr | 64.07 |
| Ps0013 | speckled kidney bean | 16.37 | 7.14 | 7.62 | 4.46 | Tr | Tr | 35.61 |
| Ps0014 | giant white kidney bean | 21.93 | 3.33 | 3.80 | 3.95 | Tr | Tr | 33.01 |
| Ps0015 | adzuki bean | 9.36 | 4.92 | 3.74 | 5.81 | Tr | Tr | 23.56 |
| Ps0016 | speckled lima bean | 13.29 | 4.46 | 2.48 | 2.32 | Tr | Tr | 22.66 |
| Ps0017 | tofu shreds | 50.99 | 19.63 | 14.66 | Tr | 1.16 | Tr | 86.40 |
| Ps0018 | soy milk powder | 26.81 | 8.91 | 5.98 | 4.84 | 2.08 | Tr | 48.62 |
| Ps0019 | soft tofu (southern style) | 21.53 | 8.85 | 5.43 | 1.42 | Tr | Tr | 37.24 |
| Ps0020 | firm tofu (northern style) | 15.17 | 5.97 | 4.30 | 2.72 | 1.08 | Tr | 29.23 |
| Ps0021 | soy milk | 4.08 | 1.76 | 1.24 | Tr | Tr | Tr | 7.08 |
|  | **Vegetables** |  |  |  |  |  |  |  |
| Ps0022 | green pea | 55.20 | 7.45 | 5.33 | 4.72 | Tr | Tr | 72.70 |
| Ps0023 | lettuce romaine / cos lettuce | 16.68 | 3.31 | 7.91 | 3.09 | 0.16 | Tr | 31.15 |
| Ps0024 | baby bok choy | 8.48 | 1.41 | Tr | 0.26 | Tr | Tr | 10.15 |
| Ps0025 | mini Chinese cabbage | 12.16 | 0.26 | 2.50 | 0.15 | Tr | Tr | 15.07 |
| Ps0026 | round lettuce | 10.45 | 1.29 | 4.31 | 1.34 | Tr | Tr | 17.39 |
| Ps0027 | green cabbage | 11.61 | 2.01 | 10.37 | Tr | Tr | Tr | 13.62 |
| Ps0028 | spinach | 5.37 | 0.78 | 2.90 | 0.76 | 0.71 | Tr | 10.51 |
| Ps0029 | garlic chives | 11.92 | 0.60 | Tr | 0.53 | Tr | Tr | 13.05 |
| Ps0030 | Chinese Napa cabbage | 9.65 | 0.54 | 2.10 | 0.50 | Tr | Tr | 12.52 |
| Ps0031 | carrot | 13.97 | 1.94 | 2.99 | 0.23 | 0.16 | Tr | 19.29 |
| Ps0032 | coriander / cilantro | 9.26 | 1.14 | 7.94 | 0.36 | Tr | Tr | 18.70 |
| Ps0033 | snow pea | 14.02 | 1.44 | 2.00 | 0.26 | Tr | Tr | 17.73 |
| Ps0034 | yard-long bean | 8.60 | 1.39 | 3.88 | 0.72 | Tr | Tr | 14.59 |
| Ps0035 | cowpea | 19.40 | 3.76 | 6.05 | 0.56 | Tr | Tr | 29.77 |
| Ps0036 | soybean sprout | 7.52 | 1.82 | 5.44 | 0.48 | Tr | Tr | 15.26 |
| Ps0037 | cauliflower | 40.79 | 0.88 | 1.12 | Tr | Tr | Tr | 42.79 |
| Ps0038 | broccoli | 34.48 | 5.31 | 0.74 | 0.44 | Tr | Tr | 40.96 |
| Ps0039 | celery | 8.87 | 0.50 | 4.20 | 0.47 | 0.08 | Tr | 14.11 |
| Ps0040 | lotus root | 8.45 | 0.84 | 0.37 | 0.56 | Tr | Tr | 10.23 |
| Ps0041 | tomato | 2.94 | 0.62 | 1.88 | 0.73 | Tr | Tr | 6.17 |
| Ps0042 | cucumber | 3.75 | 0.17 | 2.90 | 0.28 | 0.11 | Tr | 7.20 |
| Ps0043 | zucchini | 7.31 | 0.42 | 8.40 | 0.41 | 0.17 | Tr | 16.69 |
| Ps0044 | white radish | 4.28 | 1.36 | 0.09 | Tr | 0.05 | Tr | 5.77 |
| Ps0045 | bell pepper (green) | 2.11 | 1.07 | Tr | Tr | Tr | Tr | 3.18 |
| Ps0046 | eggplant | 2.01 | 0.23 | 0.65 | 0.14 | Tr | Tr | 3.03 |
| Ps0047 | wax gourd / winter melon | 0.83 | 0.13 | 0.24 | - | Tr | Tr | 1.20 |
| Ps0048 | green chili | 8.08 | 2.75 | 0.82 | 1.12 | 0.36 | Tr | 13.13 |
| Ps0049 | onion | 6.58 | 0.44 | 1.33 | 0.25 | Tr | Tr | 8.60 |
| Ps0050 | ginger | 9.88 | 1.38 | 1.48 | 1.95 | 0.25 | Tr | 14.94 |
| Ps0051 | garlic scape | 10.55 | 1.33 | 0.60 | Tr | Tr | Tr | 12.48 |
| Ps0052 | garlic | 8.71 | 1.99 | 0.46 | Tr | Tr | Tr | 11.15 |
| Ps0053 | green onion | 16.19 | 5.14 | Tr | 0.74 | Tr | Tr | 22.07 |
|  | **Fruits** |  |  |  |  |  |  |  |
| Ps054 | navel orange | 27.02 | 3.50 | 1.62 | 0.50 | Tr | Tr | 32.63 |
| Ps055 | orange | 19.64 | 2.84 | 1.28 | 0.53 | Tr | Tr | 24.30 |
| Ps056 | mandarin / tangerine | 21.14 | 2.49 | 1.36 | 0.54 | Tr | Tr | 25.53 |
| Ps057 | mango | 19.43 | 2.73 | 1.47 | 0.81 | Tr | Tr | 24.44 |
| Ps058 | Xinjiang Gong pear | 17.26 | 0.31 | 0.19 | 0.15 | Tr | Tr | 17.91 |
| Ps059 | Xinjiang fragrant pear | 15.64 | 0.18 | 0.42 | 0.22 | Tr | Tr | 16.45 |
| Ps060 | Ya pear | 11.34 | 0.24 | 0.20 | 0.94 | Tr | Tr | 12.73 |
| Ps061 | snowflake pear | 7.99 | 0.21 | 0.26 | 0.18 | Tr | Tr | 8.63 |
| Ps062 | kiwifruit | 13.40 | 1.11 | 1.95 | 1.00 | Tr | Tr | 17.47 |
| Ps063 | papaya | 8.63 | 5.35 | 2.87 | Tr | Tr | Tr | 16.86 |
| Ps064 | pineapple | 12.04 | 3.96 | 0.22 | 0.44 | Tr | Tr | 16.66 |
| Ps065 | grape | 12.32 | 1.21 | 0.21 | 1.09 | Tr | Tr | 14.83 |
| Ps066 | cherry tomato | 7.97 | 1.42 | 3.42 | 1.56 | Tr | Tr | 14.37 |
| Ps067 | peach | 11.56 | 0.46 | 1.64 | Tr | Tr | Tr | 13.66 |
|  | **Vegetable Oils** |  |  |  |  |  |  |  |
| Ps068 | peanut oil | 175.44 | 37.12 | 23.83 | 23.30 | Tr | Tr | 259.70 |
| Ps069 | soybean oil | 180.90 | 60.72 | 58.34 | 15.92 | 1.82 | Tr | 317.12 |
| Ps070 | rapeseed oil / canola oil | 341.50 | 155.01 | 8.04 | 11.36 | 3.69 | 53.02 | 570.16 |
| Ps071 | sesame oil | 370.57 | 109.74 | 42.36 | 65.82 | Tr | Tr | 588.48 |
| Ps072 | olive oil | 216.36 | 10.58 | 4.07 | 39.10 | Tr | Tr | 270.10 |
| Ps073 | linseed oil / flaxseed oil | 237.50 | 112.06 | 53.53 | 29.59 | Tr | 9.33 | 441.83 |
| Ps074 | sunflower oil | 268.00 | 53.51 | 31.81 | 18.95 | Tr | Tr | 372.26 |
| Ps075 | safflower oil | 168.34 | 50.00 | 25.43 | 23.29 | 3.39 | Tr | 270.45 |
| Ps076 | Camellia seed oil | 52.06 | 21.52 | 14.14 | 30.20 | Tr | Tr | 87.72 |
| Ps077 | tea-seed oil | 38.72 | 13.04 | 11.91 | 116.97 | Tr | Tr | 180.63 |
|  |  |  |  |  |  |  |  |  |

Note: “ Tr ” indicates that the compound was either not detected.

**Table S2**. Major food sources of phytosterols included in the Food frequency questionnaire

| **Food Groups** | **Main sources of food** |
| --- | --- |
| Rice and rice products | Rice, rice noodles |
| Wheat flour and wheat products | Steamed buns/rolls/pancakes, noodles, instant noodles, wontons/dumplings, baozi/jiaozi (stuffed buns/dumplings), bread, all types of porridge |
| Coarse grains and cereals | Millet, corn, sorghum, mung beans, red beans |
| Fried flour products | Deep-fried dough sticks (youtiao) |
| Mushrooms and fungi | Enoki mushrooms, black fungus, shiitake mushrooms, oyster mushrooms |
| Vegetables | Chinese cabbage, cabbage, spinach, tomatoes, cucumber, winter melon, celery, eggplant, broccoli, cauliflower, carrots, white radish, bell peppers (green/red), chili peppers (green/red), Chinese chives, onions, garlic shoots, lotus root, scallions/green onions, garlic, ginger |
| Legumes and legume products | Soybeans, tofu, dried tofu, soft tofu, tofu curds, soy milk, dried bean curd sticks, bean curd sheets |
| Fruits | Pears, tangerines/oranges, peaches, pineapple, kiwi, fresh grapes, all other types of fruits |
| Nuts and seeds | Sunflower/pumpkin seeds, peanuts, walnuts, pistachios, hazelnuts |
| Vegetable oils | All commonly available commercial vegetable oils |

Dietary phytosterol intake was calculated based on data from the China Food Composition Table (6th Edition) combined with participants' reported daily food consumption. Individual phytosterol intakes were quantified, including β-sitosterol (mg), campesterol (mg), stigmasterol (mg), β-sitostanol (mg) and campestanol (mg). Total phytosterol intake was defined as the sum of these five phytosterol components.

**Table S3:** Feed ingredient content for animal experiment

|  | **Normal control group** | | **Model group** | |
| --- | --- | --- | --- | --- |
|  | gm | kcal | gm | kcal |
| Protein (%) | 19.24 | 20.11 | 22.46 | 20.12 |
| Carbohydrate (%) | 42.75 | 69.85 | 44.36 | 39.74 |
| Fat (%) | 4.27 | 10.03 | 19.91 | 40.14 |
| Total (%) |  | 100.00 |  | 100.00 |
| kcal/gm | 3.83 |  | 4.46 |  |
|  |  |  |  |  |
| Ingredient | gm | kcal | gm | kcal |
| Casein, 80 mesh | 200 | 800 | 200 | 800 |
| L-Cystine | 3 | 12 | 3 | 12 |
|  |  |  |  |  |
| Corn Starch | 350 | 1400 | 0 | 0 |
| Dextrose, Monohydrate | 169 | 676 |  |  |
| Fructose |  |  | 200 | 800 |
| Maltodextrin 2 | 85 | 340 | 100 | 400 |
| Sucrose | 100 | 400 | 100 | 400 |
|  |  |  |  |  |
| Cellulose, BW200 | 50 | 0 | 50 | 0 |
|  |  |  |  |  |
| Soybean Oil | 45 | 405 |  |  |
| Lard |  |  | 180 | 1620 |
|  |  |  |  |  |
| Mineral Mix S10026B | 50 | 0 | 50 | 0 |
| Vitamin mix, V10001C | 1 | 4 | 1 | 4 |
|  |  |  |  |  |
| Choline Bitartrate | 2 | 0 | 2 | 0 |
| Cholesterol |  |  | 18 | 0 |
|  |  |  |  |  |
| Total | 1055 | 4037 | 904 | 4036 |

All feed processing is entrusted to Jiangsu Synergy Pharmaceutical and Biological Engineering Co., Ltd. to complete the production (Feed Production License: Su Feeding Certificate (2024)01008). Mixed Minerals S10026B includes sucrose particles (179.82g/1000g), potassium citrate hydrate (330.00g/1000g), calcium phosphate (260.00g/1000g), calcium carbonate (110.00g/1000g), sodium chloride (51.80g/1000g), magnesium sulphate heptahydrate (51.52g/1000g), magnesium oxide (8.38g/1000g). grams/1000 grams), magnesium oxide (8.38 grams/1000 grams), iron citrate (4.20 grams/1000 grams), manganese carbonate hydrate (2.45 grams/1000 grams), zinc carbonate (1.12 grams/1000 grams), potassium chromium sulfate (0.39 grams/1000 grams), copper carbonate (0.21 grams/1000 grams), ammonium molybdate tetrahydrate (0.06 grams/1000 grams), sodium fluoride (0.00 grams/1000 grams) 1000 g), sodium fluoride (0.04 g/1000 g), sodium selenite (0.01 g/1000 g) and potassium iodate (0.01 g/1000 g). Mixed Vitamin V10001C specifically includes sucrose particles (78.42 g/100 g), 50% vitamin E acetate (10.00 g/100 g), Niacin (3.00 g/100 g), 1% biotin (2.00 g/100 g), D-pantothenic acid semicalcium salt (vitamin B5) (1.60 g/100 g), 100,000 IU/gm of vitamin D3 (1.00 g/100 g), 0.1% Vitamin B12 (1.00 g/100 g), 500,000 IU/gm of retinyl acetate (0.80 g/100 g), pyridoxine hydrochloride (vitamin B6) (0.70 g/100 g), riboflavin (0.60 g/100 g ), Thiamine Hydrochloride (Vitamin B1) (0.60 g/100 g), Folic Acid (0.20 g/100 g), and Menadione Bisulfite (0.08 g/100 g).

**Table S4**. Baseline characteristics of participants with dyslipidemia and Healthy Controls

| **Variables** | **All Participants**  **(*n* = 183)** | **Patients with dyslipidemia**  **(*n* = 85)** | **Healthy Controls**  **(*n* = 98)** | ***P*** |
| --- | --- | --- | --- | --- |
| Age (years) ^a^ | 44.28±10.18 | 46.08±9.50 | 42.72±10.54 | 0.026 |
| Age categories (years) ^b^ |  |  |  |  |
| <40 | 54 (29.50) | 21 (24.70) | 33 (33.70) | 0.215 |
| 40-50 | 73 (39.90) | 33 (38.80) | 40 (40.80) |  |
| >50 | 56 (30.60) | 31 (36.50) | 25 (25.50) |  |
| Sex ^b^ |  |  |  |  |
| Female | 85 (46.40) | 33 (38.80) | 52 (53.10) | 0.054 |
| Male | 98 (53.60) | 52 (61.20) | 46 (46.90) |  |
| Education level ^b^ |  |  |  |  |
| Bachelor's degree or lower | 171 (93.40) | 81 (47.40) | 90 (52.60) | 0.346 |
| Graduate degree or higher | 12 (6.60) | 4 (33.30) | 8 (66.70) |  |
| BMI (kg/m^2^) ^a^ | 24.11±3.56 | 25.07±3.93 | 23.29±2.99 | ＜0.001 |
| BMI categories (kg/m^2^) ^b^ |  |  |  |  |
| ≤23.9 | 93 (50.80) | 35 (41.20) | 58 (59.20) | 0.015 |
| >23.9 | 90 (49.20) | 50 (58.80) | 40 (40.80) |  |
| Total phytosterol intake (mg/day) ^a^ | 230.13±64.35 | 226.99±58.66 | 232.84±69.09 | 0.541 |
| Total energy intake (kcal/day) ^a^ | 1811.93±380.52 | 1795.00±404.26 | 1826.61±360.13 | 0.577 |
| Smoking status ^b^ |  |  |  |  |
| No | 141 (77.00) | 58 (68.20) | 83 (84.70) | 0.008 |
| Yes | 42 (23.00) | 27 (31.80) | 15 (15.30) |  |
| Alcohol consumption ^b^ |  |  |  |  |
| No | 93 (50.80) | 41 (48.20) | 52 (53.10) | 0.515 |
| Yes | 90 (49.20) | 44 (51.80) | 46 (46.90) |  |
| Physical activity level  (MET-min/week) ^a^ | 2398.16±857.18 | 2314.99±896.36 | 2470.30±819.48 | 0.223 |
| TC (mmol/L) ^a^ | 4.95±1.13 | 5.59±1.28 | 4.39±0.57 | ＜0.001 |
| TG (mmol/L) ^d^ | 1.16 (0.78, 1.78) | 1.90 (1.04, 3.31) | 0.89 (0.66, 1.24) | ＜0.001 |
| HDL-C (mmol/L) ^a^ | 1.47±0.45 | 1.47±0.57 | 1.46±0.31 | 0.953 |
| LDL-C (mmol/L) ^a^ | 2.83±0.90 | 3.15±1.11 | 2.55±0.52 | ＜0.001 |
| Hypertension ^b^ |  |  |  |  |
| No | 151 (82.50) | 67 (78.80) | 84 (85.70) | 0.221 |
| Yes | 32 (17.50) | 18 (21.20) | 14 (14.30) |  |
| Diabetes ^c^ |  |  |  |  |
| No | 177 (96.70) | 81 (95.30) | 96 (98.00) | 0.553 |
| Yes | 6 (3.30) | 4 (4.70) | 2 (2.00) |  |
| Chronic disease ^b^ |  |  |  |  |
| No | 149 (81.40) | 65 (76.50) | 84 (85.70) | 0.109 |
| Yes | 34 (18.60) | 20 (23.50) | 14 (14.30) |  |
|  |  |  |  |  |

^a^ Continuous variables are presented as mean ± SD and were compared using independent samples t-test; ^b^ Categorical variables are presented as n (%) and were compared using Pearson's chi-square test; ^c^ using chi-square test with continuity correction; ^d^ non-normally distributed variables are presented as median (interquartile range) and were compared using Mann-Whitney U test.

**Figure S1**. Stacked-bar plots of the top 12 most abundant taxa at different classification levels. A: Phylum level; B: Genus level. LPS_baseline, Low-phytosterol diet group at the baseline; LPS_endpoint, Low-phytosterol diet group at the three months; HPS_baseline, High-phytosterol diet group at the baseline; HPS_endpoint, High-phytosterol diet group at the three months.

**Table S5**. LMM analysis of genus-level gut microbiota shifts following high-PS diet intervention in dyslipidemic subjects

| **genus** | **LPS group** | **HPS group** | **Model 1** | |  | **Model 2** | |
| --- | --- | --- | --- | --- | --- | --- | --- |
|  |  |  | **LS mean differences（95% CI）^a^** | ***P*** |  | **LS mean differences（95% CI）^a^** | ***P*** |
| *Arenimonas* |  |  |  |  |  |  |  |
| Baseline | 0.00 (0.00, 0.23) | 0.40 (0.00, 1.36) | -1.74 (-2.50~-0.98) | ＜0.001 |  | -1.71 (-2.46~-0.95) | ＜0.001 |
| Three months | 0.00 (0.00, 0.00) | 0.00 (0.00, 0.00) |  |  |  |  |  |
| *Cetobacterium* |  |  |  |  |  |  |  |
| Baseline | 0.52 (0.14, 1.13) | 1.15 (0.24, 2.87) | -1.56 (-2.69~-0.43) | 0.008 |  | -1.61 (-2.75~-0.48) | 0.007 |
| Three months | 0.00 (0.00, 0.05) | 0.00 (0.00, 0.02) |  |  |  |  |  |
| *Defluviitoga* |  |  |  |  |  |  |  |
| Baseline | 3.40 (0.62, 5.80) | 0.13 (0.00, 1.57) | 1.99 (0.86~3.13) | ＜0.001 |  | 1.99 (0.88~3.10) | ＜0.001 |
| Three months | 0.01 (0.00, 0.07) | 0.00 (0.00, 0.05) |  |  |  |  |  |
| *Dietzia* |  |  |  |  |  |  |  |
| Baseline | 0.75 (0.00, 2.29) | 1.37 (0.36, 3.03) | -1.28 (-2.41~-0.15) | 0.028 |  | -1.23 (-2.37~-0.10) | 0.035 |
| Three months | 0.00 (0.00, 0.03) | 0.00 (0.00, 0.02) |  |  |  |  |  |
| *Limosilactobacillus* |  |  |  |  |  |  |  |
| Baseline | 0.00 (0.00, 0.11) | 0.00 (0.00, 0.25) | -1.04 (-1.98~-0.10) | 0.034 |  | -1.02 (-1.97~-0.08) | 0.037 |
| Three months | 0.00 (0.00, 0.08) | 0.00 (0.00, 0.00) |  |  |  |  |  |
| *Mycoplasma* |  |  |  |  |  |  |  |
| Baseline | 0.31 (0.00, 1.88) | 0.90 (0.19, 1.87) | -1.20 (-2.31~-0.09) | 0.036 |  | -1.13 (-2.23~-0.03) | 0.046 |
| Three months | 0.00 (0.00, 0.02) | 0.00 (0.00, 0.03) |  |  |  |  |  |
| *Opitutus* |  |  |  |  |  |  |  |
| Baseline | 0.02 (0.00, 0.69) | 0.48 (0.05, 1.26) | -1.42 (-2.42~-0.42) | 0.007 |  | -1.39 (-2.40~-0.38) | 0.008 |
| Three months | 0.00 (0.00, 0.00) | 0.00 (0.00, 0.00) |  |  |  |  |  |

Notes: Values for all bacterial genera are presented as ×10⁻³. Only taxonomically defined genera achieving *P* < 0.05 in linear mixed-effects model (LMM) analyses are listed; unclassified taxa were excluded. Genera with detection rates >20% at any study group or time point were included in statistical modelling. LMM was applied to assess longitudinal shifts in genus-level gut microbiota. ^a^ Least-squares (LS) mean differences between groups on the centered log‑ratio (CLR) scale. Model 1: unadjusted; Model 2: adjusted for age, sex and baseline BMI.

**Table S6**. LMM analysis for BSH-active genus abundance changes upon high-PS diet intervention in dyslipidemic patients

| **genus** | **LPS group** | **HPS group** | **Model 1** | |  | **Model 2** | |
| --- | --- | --- | --- | --- | --- | --- | --- |
|  |  |  | **LS mean differences（95% CI）^a^** | ***P*** |  | **LS mean differences（95% CI）^a^** | ***P*** |
| *Bifidobacterium* |  |  |  |  |  |  |  |
| Baseline | 1.55 (0.33, 2.65) | 2.56 (1.01, 4.11) | -0.12 (-1.67~1.43) | 0.877 |  | -0.12 (-1.68~1.44) | 0.880 |
| Three months | 36.36 (1.19, 92.21) | 40.60 (10.71, 112.86) |  |  |  |  |  |
| *Alistipes* |  |  |  |  |  |  |  |
| Baseline | 2.93 (0.88, 6.11) | 2.74 (1.27, 3.93) | 0.55 (-1.19~2.28) | 0.540 |  | 0.49 (-1.23~2.20) | 0.581 |
| Three months | 0.28 (0.04, 1.92) | 1.07 (0.12, 9.53) |  |  |  |  |  |
| *Bacteroides* |  |  |  |  |  |  |  |
| Baseline | 10.69 (7.13, 78.98) | 11.54 (8.08, 29.96) | 1.07 (-0.18~2.33) | 0.096 |  | 1.10 (-0.15~2.36) | 0.087 |
| Three months | 4.96 (1.88, 29.07) | 25.78 (5.12, 65.95) |  |  |  |  |  |
| *Flavobacterium* |  |  |  |  |  |  |  |
| Baseline | 3.32 (1.70, 4.96) | 3.12 (2.05, 4.75) | -0.11 (-0.83~0.61) | 0.764 |  | -0.10 (-0.81~0.62) | 0.795 |
| Three months | 0.04 (0.02, 0.09) | 0.05 (0.00, 0.07) |  |  |  |  |  |
| *Parabacteroides* |  |  |  |  |  |  |  |
| Baseline | 2.16 (0.41, 5.34) | 1.82 (0.71, 3.38) | 0.91 (-0.71~2.53) | 0.276 |  | 0.88 (-0.72~2.48) | 0.283 |
| Three months | 0.28 (0.09, 1.38) | 1.25 (0.31, 5.83) |  |  |  |  |  |
| *Bacillus* |  |  |  |  |  |  |  |
| Baseline | 7.68 (4.61, 11.61) | 7.38 (5.14, 11.89) | -0.27 (-1.09~0.56) | 0.527 |  | -0.20 (-1.04~0.63) | 0.635 |
| Three months | 0.10 (0.05, 0.28) | 0.12 (0.00, 0.37) |  |  |  |  |  |
| *Enterococcus* |  |  |  |  |  |  |  |
| Baseline | 1.38 (0.22, 2.45) | 1.02 (0.42, 1.94) | -0.58 (-1.77~0.61) | 0.346 |  | -0.60 (-1.78~0.58) | 0.326 |
| Three months | 0.26 (0.02, 1.32) | 0.09 (0.00, 1.00) |  |  |  |  |  |
| *Lactobacillus* |  |  |  |  |  |  |  |
| Baseline | 9.00 (4.19, 13.45) | 7.25 (3.68, 11.40) | -0.04 (-0.98~0.91) | 0.940 |  | 0.01 (-0.93~0.95) | 0.986 |
| Three months | 0.19 (0.05, 0.39) | 0.05 (0.00, 0.34) |  |  |  |  |  |
| *Streptococcus* |  |  |  |  |  |  |  |
| Baseline | 1.64 (0.94, 4.32) | 1.57 (0.81, 3.16) | -0.09 (-1.02~0.83) | 0.843 |  | -0.08 (-0.99~0.84) | 0.869 |
| Three months | 31.58 (20.66, 74.01) | 29.66 (12.80, 58.83) |  |  |  |  |  |
| *Blautia* |  |  |  |  |  |  |  |
| Baseline | 1.42 (0.62, 4.24) | 1.94 (0.58, 3.39) | -0.21 (-1.19~0.78) | 0.685 |  | -0.18 (-1.17~0.80) | 0.721 |
| Three months | 110.54 (53.02, 210.39) | 121.20 (53.51, 178.44) |  |  |  |  |  |
| *Lachnoclostridium* |  |  |  |  |  |  |  |
| Baseline | 0.46 (0.00, 2.17) | 0.64 (0.14, 1.22) | -0.93 (-2.60~0.74) | 0.278 |  | -0.85 (-2.51~0.80) | 0.316 |
| Three months | 0.12 (0.00, 1.33) | 0.08 (0.00, 0.93) |  |  |  |  |  |
| *Faecalibacterium* |  |  |  |  |  |  |  |
| Baseline | 3.81 (0.24, 41.62) | 2.63 (0.81, 82.07) | -0.01 (-1.85~1.84) | 0.993 |  | -0.05 (-1.88~1.78) | 0.957 |
| Three months | 10.56 (1.60, 39.05) | 21.75 (3.18, 66.68) |  |  |  |  |  |
| *Ruminococcus* |  |  |  |  |  |  |  |
| Baseline | 1.14 (0.00, 2.09) | 0.43 (0.12, 2.28) | -1.51 (-3.42~0.40) | 0.126 |  | -1.53 (-3.45~0.40) | 0.126 |
| Three months | 2.54 (0.21, 22.02) | 0.79 (0.00, 6.30) |  |  |  |  |  |
| *Turicibacter* |  |  |  |  |  |  |  |
| Baseline | 1.68 (0.82, 2.89) | 1.76 (1.19, 2.74) | -0.10 (-1.41~1.21) | 0.880 |  | -0.07 (-1.39~1.25) | 0.918 |
| Three months | 0.16 (0.05, 0.85) | 0.17 (0.04, 1.21) |  |  |  |  |  |
| *Pseudomonas* |  |  |  |  |  |  |  |
| Baseline | 6.62 (3.51, 9.10) | 5.13 (3.25, 7.83) | -0.08 (-0.84~0.67) | 0.829 |  | -0.06 (-0.82~0.69) | 0.870 |
| Three months | 0.07 (0.02, 0.21) | 0.04 (0.00, 0.13) |  |  |  |  |  |

Notes: Data for all bacterial genera are presented as ×10⁻³. Only genera with detection frequencies >20% across all study groups and sampling time points were included for analysis. Linear mixed-effects model (LMM) was used to evaluate longitudinal alterations in the relative abundance of bile salt hydrolase (BSH)-active bacterial genera at the genus level. ^a^ Least-squares (LS) mean differences between the two groups on the centered log‑ratio (CLR) scale. Model 1, unadjusted; Model 2, adjusted for age, sex and baseline BMI.

**Table S7**. Comparison of serum primary bile acid levels between the two groups before and after intervention

|  | **Baseline** | **Three months** | **Model 1** | | |  | **Model 2** | | |
| --- | --- | --- | --- | --- | --- | --- | --- | --- | --- |
|  |  |  | ***P*_group_** | ***P*_time_** | ***P*_interaction_** |  | ***P*_group_** | ***P*_time_** | ***P*_interaction_** |
| CDCA (nmol/L) |  |  |  |  |  |  |  |  |  |
| LPS group | 419.59 (147.47, 886.27) | 503.20 (192.79, 965.38) | 0.031 | 0.471 | 0.003 |  | 0.055 | 0.471 | 0.003 |
| HPS group | 295.91 (184.88, 807.64) | 201.73 (80.35, 518.98) |  |  |  |  |  |  |  |
| HCA (nmol/L) |  |  |  |  |  |  |  |  |  |
| LPS group | 2.81 (0.004, 10.79) | 5.88 (0.004, 16.37) | 0.766 | 0.791 | 0.418 |  | 0.486 | 0.791 | 0.418 |
| HPS group | 4.54 (0.004, 17.97) | 5.78 (0.003, 10.84) |  |  |  |  |  |  |  |
| CA (nmol/L) |  |  |  |  |  |  |  |  |  |
| LPS group | 49.43 (28.80, 114.45) | 78.77 (46.61, 151.87) | 0.312 | 0.167 | ＜0.001 |  | 0.350 | 0.167 | ＜0.001 |
| HPS group | 64.73 (44.42, 190.24) | 36.76 (14.92, 87.32) |  |  |  |  |  |  |  |
| GCDCA (nmol/L) |  |  |  |  |  |  |  |  |  |
| LPS group | 515.23 (245.38, 782.21) | 588.06 (367.73, 1230.91) | 0.028 | 0.076 | 0.104 |  | 0.057 | 0.076 | 0.104 |
| HPS group | 285.70 (190.16, 661.29) | 376.64 (206.97, 666.10) |  |  |  |  |  |  |  |
| GHCA (nmol/L) |  |  |  |  |  |  |  |  |  |
| LPS group | 0.004 (0.003, 0.01) | 6.47 (0.004, 15.55) | 0.102 | ＜0.001 | 0.097 |  | 0.134 | ＜0.001 | 0.097 |
| HPS group | 0.004 (0.003, 1.88) | 10.03 (5.57, 18.32) |  |  |  |  |  |  |  |
| TCDCA (nmol/L) |  |  |  |  |  |  |  |  |  |
| LPS group | 51.44 (33.20, 88.61) | 67.49 (25.77, 113.85) | 0.472 | 0.154 | 0.473 |  | 0.339 | 0.154 | 0.473 |
| HPS group | 42.30 (20.90, 67.14) | 49.33 (23.31, 94.43) |  |  |  |  |  |  |  |
| TCA (nmol/L) |  |  |  |  |  |  |  |  |  |
| LPS group | 15.41 (6.65, 25.49) | 11.28 (5.35, 29.01) | 0.991 | 0.021 | 0.458 |  | 0.630 | 0.021 | 0.458 |
| HPS group | 11.14 (6.27, 23.41) | 10.43 (1.89, 27.17) |  |  |  |  |  |  |  |
| GCDCA-3S (nmol/L) |  |  |  |  |  |  |  |  |  |
| LPS group | 74.01 (29.95, 178.14) | 61.59 (43.66, 126.34) | 0.697 | 0.019 | 0.490 |  | 0.692 | 0.019 | 0.490 |
| HPS group | 89.06 (28.57, 118.30) | 59.72 (44.94, 104.97) |  |  |  |  |  |  |  |
| CDCA-3Gln (nmol/L) |  |  |  |  |  |  |  |  |  |
| LPS group | 150.81 (127.83, 189.63) | 19.52 (0.004, 119.74) | 0.009 | ＜0.001 | 0.234 |  | 0.018 | ＜0.001 | 0.234 |
| HPS group | 134.87 (0.004, 168.85) | 0.005 (0.002, 16.46) |  |  |  |  |  |  |  |
| GCDCA-3Gln (nmol/L) |  |  |  |  |  |  |  |  |  |
| LPS group | 76.76 (39.86, 122.08) | 53.25 (42.32, 99.71) | 0.080 | 0.018 | 0.111 |  | 0.028 | 0.018 | 0.111 |
| HPS group | 49.52 (26.29, 87.34) | 46.39 (24.27, 77.50) |  |  |  |  |  |  |  |

Note: Given the right-skewed distribution of serum bile acid concentrations, raw values are presented as median (P_25_, P_75_). Between-group differences in bile acid levels before and after intervention were analyzed using GEE models. As the raw bile acid data were not normally distributed, all bile acid concentrations were transformed using ln(x + 1) before analysis. Model 1 was unadjusted, and Model 2 was adjusted for age, sex, and baseline BMI.

**Table S8**. Comparison of serum primary bile acids between the two groups at 3 months after intervention

|  | **Model 1** | |  | **Model 2** | |
| --- | --- | --- | --- | --- | --- |
|  | **Exponentiated coefficient (95% CI) ^a^** | ***P*** |  | **Exponentiated coefficient (95% CI) ^a^** | ***P*** |
| CDCA (nmol/L) |  |  |  |  |  |
| HPS group vs LPS group | 0.45 (0.28~0.72) | ＜0.001 |  | 0.47 (0.29~0.75) | 0.002 |
| HCA (nmol/L) |  |  |  |  |  |
| HPS group vs LPS group | 1.06 (0.70~1.61) | 0.766 |  | 1.16 (0.76~1.77) | 0.486 |
| CA (nmol/L) |  |  |  |  |  |
| HPS group vs LPS group | 0.48 (0.31~0.76) | 0.002 |  | 0.49 (0.30~0.78) | 0.003 |
| GCDCA (nmol/L) |  |  |  |  |  |
| HPS group vs LPS group | 0.69 (0.50~0.96) | 0.028 |  | 0.73 (0.52~1.01) | 0.057 |
| GHCA (nmol/L) |  |  |  |  |  |
| HPS group vs LPS group | 1.37 (0.94~2.00) | 0.102 |  | 1.36 (0.91~2.02) | 0.134 |
| TCDCA (nmol/L) |  |  |  |  |  |
| HPS group vs LPS group | 0.88 (0.63~1.23) | 0.472 |  | 0.85 (0.61~1.19) | 0.339 |
| TCA (nmol/L) |  |  |  |  |  |
| HPS group vs LPS group | 1.00 (0.60~1.65) | 0.991 |  | 0.88 (0.53~1.48) | 0.630 |
| GCDCA-3S (nmol/L) |  |  |  |  |  |
| HPS group vs LPS group | 1.09 (0.71~1.68) | 0.697 |  | 1.09 (0.71~1.67) | 0.692 |
| CDCA-3Gln (nmol/L) |  |  |  |  |  |
| HPS group vs LPS group | 0.39 (0.20~0.79) | 0.009 |  | 0.43 (0.22~0.87) | 0.018 |
| GCDCA-3Gln (nmol/L) |  |  |  |  |  |
| HPS group vs LPS group | 0.63 (0.37~1.06) | 0.080 |  | 0.62 (0.41~0.95) | 0.028 |

Note: Between-group differences in bile acid levels after intervention were analyzed using GEE models. The LPS group served as the reference group, and effect sizes are expressed as exponentiated coefficients with 95% confidence intervals (95% CIs). ^a^ Values were calculated by exponentiating the mean differences derived from ln(x + 1)-transformed data. Model 1 was unadjusted; Model 2 was adjusted for age, sex, and baseline BMI. Post hoc pairwise comparisons were conducted using the least significant difference (LSD) method.

**Table S9**. Comparison of serum secondary bile acids between the two groups before and after intervention

|  | **Baseline** | **Three months** | **Model 1** | | |  | **Model 2** | | |
| --- | --- | --- | --- | --- | --- | --- | --- | --- | --- |
|  |  |  | ***P*_group_** | ***P*_time_** | ***P*_interaction_** |  | ***P*_group_** | ***P*_time_** | ***P*_interaction_** |
| isoLCA (nmol/L) |  |  |  |  |  |  |  |  |  |
| LPS group | 0.01 (0.003, 7.55) | 0.004 (0.003, 5.88) | 0.632 | 0.246 | 0.121 |  | 0.513 | 0.246 | 0.121 |
| HPS group | 0.004 (0.003, 8.29) | 0.49 (0.003, 11.27) |  |  |  |  |  |  |  |
| LCA (nmol/L) |  |  |  |  |  |  |  |  |  |
| LPS group | 7.00 (0.004, 15.60) | 1.07 (0.003, 10.19) | 0.886 | 0.074 | 0.181 |  | 0.811 | 0.074 | 0.181 |
| HPS group | 5.57 (0.004, 9.07) | 3.70 (0.004, 10.54) |  |  |  |  |  |  |  |
| 6-ketoLCA (nmol/L) |  |  |  |  |  |  |  |  |  |
| LPS group | 0.30 (0.004, 1.52) | 0.004 (0.002, 0.005) | 0.346 | ＜0.001 | 0.605 |  | 0.666 | ＜0.001 | 0.605 |
| HPS group | 0.005 (0.003, 1.01) | 0.003 (0.002, 0.005) |  |  |  |  |  |  |  |
| 7-ketoLCA (nmol/L) |  |  |  |  |  |  |  |  |  |
| LPS group | 14.96 (4.09, 26.45) | 10.42 (4.21, 22.76) | 0.012 | 0.114 | 0.203 |  | 0.061 | 0.114 | 0.203 |
| HPS group | 10.61 (1.87, 20.25) | 4.46 (0.004, 14.36) |  |  |  |  |  |  |  |
| 12-ketoLCA (nmol/L) |  |  |  |  |  |  |  |  |  |
| LPS group | 6.09 (0.01, 19.23) | 4.70 (0.003, 12.19) | 0.672 | ＜0.001 | 0.383 |  | 0.588 | ＜0.001 | 0.383 |
| HPS group | 7.29 (2.60, 18.23) | 4.46 (0.003, 9.92) |  |  |  |  |  |  |  |
| apoCA (nmol/L) |  |  |  |  |  |  |  |  |  |
| LPS group | 0.01 (0.003, 7.24) | 0.004 (0.003, 0.004) | 0.920 | ＜0.001 | 0.197 |  | 0.925 | ＜0.001 | 0.197 |
| HPS group | 2.32 (0.004, 4.82) | 0.003 (0.002, 0.01) |  |  |  |  |  |  |  |
| isoUDCA (nmol/L) |  |  |  |  |  |  |  |  |  |
| LPS group | 122.94 (54.58, 370.98) | 166.10 (0.01, 548.79) | 0.134 | 0.133 | 0.434 |  | 0.206 | 0.133 | 0.434 |
| HPS group | 88.85 (36.73, 181.33) | 81.64 (0.01, 298.83) |  |  |  |  |  |  |  |
| UDCA (nmol/L) |  |  |  |  |  |  |  |  |  |
| LPS group | 99.07 (52.20, 223.26) | 108.67 (46.81, 231.62) | 0.009 | 0.029 | 0.125 |  | 0.020 | 0.029 | 0.125 |
| HPS group | 73.51 (38.31, 187.20) | 50.54 (17.76, 132.72) |  |  |  |  |  |  |  |
| HDCA (nmol/L) |  |  |  |  |  |  |  |  |  |
| LPS group | 2.43 (0.004, 5.44) | 0.004 (0.003, 6.34) | 0.227 | 0.008 | 0.622 |  | 0.166 | 0.008 | 0.622 |
| HPS group | 3.72 (0.004, 7.43) | 0.01 (0.003, 5.94) |  |  |  |  |  |  |  |
| 3β-DCA (nmol/L) |  |  |  |  |  |  |  |  |  |
| LPS group | 57.22 (29.74, 118.82) | 18.45 (0.004, 43.79) | 0.314 | ＜0.001 | 0.112 |  | 0.239 | ＜0.001 | 0.112 |
| HPS group | 44.29 (25.37, 73.91) | 23.24 (2.66, 55.95) |  |  |  |  |  |  |  |
| DCA (nmol/L) |  |  |  |  |  |  |  |  |  |
| LPS group | 208.86 (40.16, 323.94) | 127.88 (12.82, 306.00) | 0.235 | 0.196 | 0.403 |  | 0.203 | 0.196 | 0.403 |
| HPS group | 254.48 (100.97, 306.20) | 148.59 (56.62, 205.60) |  |  |  |  |  |  |  |
| NCA (nmol/L) |  |  |  |  |  |  |  |  |  |
| LPS group | 2.75 (0.01, 4.59) | 1.33 (0.004, 4.26) | 0.017 | 0.006 | 0.880 |  | 0.032 | 0.006 | 0.880 |
| HPS group | 1.15 (0.004, 3.04) | 0.01 (0.003, 1.74) |  |  |  |  |  |  |  |
| GLCA (nmol/L) |  |  |  |  |  |  |  |  |  |
| LPS group | 3.59 (0.004, 12.95) | 2.52 (0.004, 11.61) | 0.596 | 0.118 | 0.617 |  | 0.544 | 0.118 | 0.617 |
| HPS group | 5.61 (2.16, 13.43) | 4.42 (0.004, 7.49) |  |  |  |  |  |  |  |
| GUDCA (nmol/L) |  |  |  |  |  |  |  |  |  |
| LPS group | 108.35 (62.88, 233.55) | 103.07 (55.33, 242.95) | 0.007 | 0.030 | 0.063 |  | 0.010 | 0.030 | 0.063 |
| HPS group | 89.68 (41.87, 154.06) | 51.61 (29.42, 115.20) |  |  |  |  |  |  |  |
| GDCA (nmol/L) |  |  |  |  |  |  |  |  |  |
| LPS group | 114.60 (30.58, 471.71) | 356.72 (179.43, 496.87) | 0.072 | ＜0.001 | 0.426 |  | 0.051 | ＜0.001 | 0.426 |
| HPS group | 151.00 (57.32, 386.14) | 374.52 (335.19, 439.11) |  |  |  |  |  |  |  |
| LCA-3S (nmol/L) |  |  |  |  |  |  |  |  |  |
| LPS group | 0.23 (0.004, 4.58) | 0.01 (0.003, 2.60) | 0.818 | 0.052 | 0.159 |  | 0.693 | 0.052 | 0.159 |
| HPS group | 1.63 (0.004, 3.12) | 0.39 (0.003, 2.36) |  |  |  |  |  |  |  |
| GCA (nmol/L) |  |  |  |  |  |  |  |  |  |
| LPS group | 123.70 (59.18, 240.57) | 160.18 (79.41, 354.23) | 0.322 | 0.003 | 0.577 |  | 0.285 | 0.003 | 0.577 |
| HPS group | 79.08 (48.26, 151.18) | 97.31 (57.34, 279.40) |  |  |  |  |  |  |  |
| TLCA (nmol/L) |  |  |  |  |  |  |  |  |  |
| LPS group | 1.78 (0.004, 2.68) | 0.004 (0.002, 0.004) | 0.858 | ＜0.001 | 0.315 |  | 0.918 | ＜0.001 | 0.315 |
| HPS group | 0.83 (0.003, 2.75) | 0.003 (0.002, 0.01) |  |  |  |  |  |  |  |
| TUDCA (nmol/L) |  |  |  |  |  |  |  |  |  |
| LPS group | 0.004 (0.003, 4.93) | 11.23 (4.24, 21.51) | 0.804 | ＜0.001 | 0.987 |  | 0.519 | ＜0.001 | 0.987 |
| HPS group | 0.004 (0.003, 6.36) | 10.14 (4.58, 20.63) |  |  |  |  |  |  |  |
| TDCA (nmol/L) |  |  |  |  |  |  |  |  |  |
| LPS group | 7.16 (1.28, 21.27) | 7.51 (0.004, 17.61) | 0.146 | 0.272 | 0.643 |  | 0.218 | 0.272 | 0.643 |
| HPS group | 12.42 (2.02, 25.49) | 11.90 (3.99, 20.71) |  |  |  |  |  |  |  |
| GLCA-3S (nmol/L) |  |  |  |  |  |  |  |  |  |
| LPS group | 118.70 (19.30, 257.72) | 90.66 (3.96, 278.48) | 0.094 | 0.924 | 0.041 |  | 0.121 | 0.924 | 0.041 |
| HPS group | 148.11 (38.15, 290.50) | 163.04 (62.13, 307.49) |  |  |  |  |  |  |  |
| GUDCA-3S (nmol/L) |  |  |  |  |  |  |  |  |  |
| LPS group | 82.82 (30.60, 192.46) | 59.27 (37.23, 102.55) | 0.011 | ＜0.001 | 0.920 |  | 0.015 | ＜0.001 | 0.920 |
| HPS group | 66.22 (23.59, 101.14) | 44.17 (13.28, 78.96) |  |  |  |  |  |  |  |
| GDCA-3S (nmol/L) |  |  |  |  |  |  |  |  |  |
| LPS group | 84.32 (3.15, 116.53) | 60.17 (0.004, 139.67) | 0.077 | 0.531 | 0.180 |  | 0.081 | 0.531 | 0.180 |
| HPS group | 98.78 (31.79, 150.49) | 92.98 (40.11, 142.19) |  |  |  |  |  |  |  |
| TLCA-3S (nmol/L) |  |  |  |  |  |  |  |  |  |
| LPS group | 26.51 (0.004, 56.36) | 9.73 (0.004, 31.86) | 0.046 | 0.023 | 0.203 |  | 0.102 | 0.023 | 0.203 |
| HPS group | 37.22 (0.01, 79.36) | 37.04 (3.89, 63.59) |  |  |  |  |  |  |  |
| GDCA-3Gln (nmol/L) |  |  |  |  |  |  |  |  |  |
| LPS group | 67.56 (0.004, 128.98) | 25.26 (0.004, 72.92) | 0.529 | ＜0.001 | 0.213 |  | 0.532 | ＜0.001 | 0.213 |
| HPS group | 58.35 (26.35, 143.08) | 27.91 (0.01, 47.50) |  |  |  |  |  |  |  |
|  |  |  |  |  |  |  |  |  |  |

Note: Given the right-skewed distribution of serum bile acid concentrations, raw values are presented as median (P_25_, P_75_). Between-group differences in bile acid levels before and after intervention were analyzed using GEE models. As the raw bile acid data were not normally distributed, all bile acid concentrations were transformed using ln(x + 1) before analysis. Model 1 was unadjusted, and Model 2 was adjusted for age, sex, and baseline BMI.

**Table S10**. Comparison of serum secondary bile acids between the two groups at 3 months after intervention

|  | **Model 1** | |  | **Model 2** | |
| --- | --- | --- | --- | --- | --- |
|  | **Exponentiated coefficient (95% CI) ^a^** | ***P*** |  | **Exponentiated coefficient (95% CI) ^a^** | ***P*** |
| isoLCA (nmol/L) |  |  |  |  |  |
| HPS group vs LPS group | 1.11 (0.73~1.66) | 0.632 |  | 1.15 (0.75~1.76) | 0.513 |
| LCA (nmol/L) |  |  |  |  |  |
| HPS group vs LPS group | 1.23 (0.73~2.07) | 0.445 |  | 1.06 (0.66~1.69) | 0.811 |
| 6-ketoLCA (nmol/L) |  |  |  |  |  |
| HPS group vs LPS group | 0.90 (0.72~1.12) | 0.346 |  | 0.95 (0.77~1.18) | 0.666 |
| 7-ketoLCA (nmol/L) |  |  |  |  |  |
| HPS group vs LPS group | 0.59 (0.40~0.89) | 0.012 |  | 0.68 (0.45~1.02) | 0.061 |
| 12-ketoLCA (nmol/L) |  |  |  |  |  |
| HPS group vs LPS group | 1.11 (0.69~1.78) | 0.672 |  | 1.13 (0.72~1.78) | 0.588 |
| apoCA (nmol/L) |  |  |  |  |  |
| HPS group vs LPS group | 0.99 (0.73~1.32) | 0.920 |  | 0.99 (0.73~1.33) | 0.925 |
| isoUDCA (nmol/L) |  |  |  |  |  |
| HPS group vs LPS group | 0.58 (0.29~1.18) | 0.134 |  | 0.63 (0.30~1.29) | 0.266 |
| UDCA (nmol/L) |  |  |  |  |  |
| HPS group vs LPS group | 0.57 (0.38~0.87) | 0.009 |  | 0.59 (0.38~0.92) | 0.020 |
| HDCA (nmol/L) |  |  |  |  |  |
| HPS group vs LPS group | 1.24 (0.87~1.76) | 0.227 |  | 1.28 (0.90~1.80) | 0.166 |
| 3β-DCA (nmol/L) |  |  |  |  |  |
| HPS group vs LPS group | 1.26 (0.80~1.98) | 0.314 |  | 1.31 (0.84~2.04) | 0.239 |
| DCA (nmol/L) |  |  |  |  |  |
| HPS group vs LPS group | 1.44 (0.79~2.65) | 0.235 |  | 1.49 (0.81~2.75) | 0.203 |
| NCA (nmol/L) |  |  |  |  |  |
| HPS group vs LPS group | 0.73 (0.56~0.95) | 0.017 |  | 0.76 (0.58~0.98) | 0.032 |
| GLCA (nmol/L) |  |  |  |  |  |
| HPS group vs LPS group | 1.14 (0.71~1.82) | 0.596 |  | 1.16 (0.72~1.85) | 0.544 |
| GUDCA (nmol/L) |  |  |  |  |  |
| HPS group vs LPS group | 0.59 (0.40~0.87) | 0.007 |  | 0.60 (0.41~0.89) | 0.010 |
| GDCA (nmol/L) |  |  |  |  |  |
| HPS group vs LPS group | 1.97 (0.94~4.13) | 0.072 |  | 2.14 (1.00~4.61) | 0.051 |
| LCA-3S (nmol/L) |  |  |  |  |  |
| HPS group vs LPS group | 1.04 (0.76~1.41) | 0.818 |  | 1.07 (0.78~1.46) | 0.693 |
| GCA (nmol/L) |  |  |  |  |  |
| HPS group vs LPS group | 0.82 (0.56~1.21) | 0.322 |  | 0.80 (0.54~1.20) | 0.285 |
| TLCA (nmol/L) |  |  |  |  |  |
| HPS group vs LPS group | 0.98 (0.82~1.18) | 0.858 |  | 1.01 (0.85~1.20) | 0.918 |
| TUDCA (nmol/L) |  |  |  |  |  |
| HPS group vs LPS group | 1.05 (0.72~1.54) | 0.804 |  | 1.13 (0.77~1.66) | 0.519 |
| TDCA (nmol/L) |  |  |  |  |  |
| HPS group vs LPS group | 1.48 (0.87~2.51) | 0.146 |  | 1.43 (0.81~2.52) | 0.218 |
| GLCA-3S (nmol/L) |  |  |  |  |  |
| HPS group vs LPS group | 2.54 (1.17~5.50) | 0.018 |  | 2.48 (1.12~5.47) | 0.025 |
| GUDCA-3S (nmol/L) |  |  |  |  |  |
| HPS group vs LPS group | 0.60 (0.40~0.89) | 0.011 |  | 0.62 (0.42~0.91) | 0.015 |
| GDCA-3S (nmol/L) |  |  |  |  |  |
| HPS group vs LPS group | 1.89 (0.93~3.83) | 0.077 |  | 1.96 (0.92~4.17) | 0.081 |
| TLCA-3S (nmol/L) |  |  |  |  |  |
| HPS group vs LPS group | 1.93 (1.01~3.70) | 0.046 |  | 1.81 (0.89~3.67) | 0.102 |
| GDCA-3Gln (nmol/L) |  |  |  |  |  |
| HPS group vs LPS group | 1.24 (0.63~2.44) | 0.529 |  | 1.26 (0.61~2.64) | 0.532 |

Note: Between-group differences in bile acid levels after intervention were analyzed using GEE models. The LPS group served as the reference group, and effect sizes are expressed as exponentiated coefficients with 95% confidence intervals (95% CIs). ^a^ Values were calculated by exponentiating the mean differences derived from ln(x + 1)-transformed data. Model 1 was unadjusted; Model 2 was adjusted for age, sex, and baseline BMI. Post hoc pairwise comparisons were conducted using the least significant difference (LSD) method.

**Figure S2**. Correlation heatmap of intervention-related changes in differential bile acids and serum lipid parameters. Correlations between pre- and post-intervention changes in each variable were assessed using Spearman rank correlation analysis. * *P* < 0.05, ** *P* < 0.01, **** P* < 0.001.

**Figure S3**. Correlation heatmap of changes in BSH-active genera and serum lipid parameters. Correlations between pre- and post-intervention changes in each variable were assessed using Spearman rank correlation analysis. * *P* < 0.05, ** *P* < 0.01.

**Figure S4**. Alpha-diversity comparisons among experimental groups. Data are presented as median and interquartile range (IQR) (*n* = 6 animals/group). Overall differences among groups were assessed using the Kruskal-Wallis H test, followed by Dunn’s test for pairwise comparisons. NCD, normal control diet; HFD, high-fat diet; HFD+PS, HFD supplemented with phytosterols.

**Figure S5**. Body weight changes at the end of FMT experiment. *n* = 7 animals/group. Data are presented as mean±SEM. Differences between groups were performed using an independent-samples t-test. ***** compared to HFD-FMT, *P* < 0.05; ****** compared to HFD-FMT, *P* < 0.01; ******* compared to HFD-FMT, *P* < 0.001.

**Figure S6**. Serum level of TG at the end of FMT experiment. *n* = 7 animals/group. Data are presented as mean±SEM. Differences between groups were performed using an independent-samples t-test. ***** compared to HFD-FMT, *P* < 0.05; ****** compared to HFD-FMT, *P* < 0.01; ******* compared to HFD-FMT, *P* < 0.001.

**Figure S7**. Original protein bands from liver tissue

**Figure S8**. Original protein bands from ileal tissue

**Figure S9**. Original protein bands from liver tissue for NCD, HFD, HFD+HDCA and HFD+THDCA groups.

**Figure S10**. Original protein bands from ileal tissue for NCD, HFD, HFD+HDCA and HFD+THDCA groups.

**Table S11**. Comparison of serum bile acid profiles between the two groups (nmol/L)

| **Bile acid** | **Placebo group** | **PS group** | ***P*** |
| --- | --- | --- | --- |
| —/isoLCA | 5.34 (0.98, 17.63) | 2.48 (1.04, 6.61) | 0.197 |
| LCA | 3.08 (0.87, 7.18) | 2.10 (0.29, 5.03) | 0.164 |
| 23norDCA | 0.46 (0.0002, 1.20) | 0.21 (0.0001, 0.97) | 0.473 |
| 6-ketoLCA | 4.35 (3.10, 6.23) | 3.91 (2.93, 5.65) | 0.869 |
| 7-ketoLCA | 28.92 (25.28, 32.74) | 28.02 (24.16, 30.59) | 0.562 |
| 12-ketoLCA | 5.45 (1.58, 17.6 8) | 4.33 (0.39, 7.41) | 0.312 |
| apoCA | 5.75 (1.93, 10.42) | 3.93 (1.63, 8.28) | 0.210 |
| isoUDCA | 235.61 (77.71, 542.10) | 214.73 (99.53, 438.78) | 0.842 |
| isoHDCA | 6.90 (3.95, 11.03) | 5.23 (3.31, 8.31) | 0.272 |
| UDCA | 94.98 (33.32, 239.85) | 101.87 (46.21, 139.14) | 0.762 |
| HDCA | 19.27 (3.75, 37.84) | 15.43 (1.32, 27.52) | 0.264 |
| 3β-DCA | 84.38 (43.29, 128.24) | 69.66 (42.16, 116.34) | 0.736 |
| CDCA | 255.19 (120.98, 495.77) | 261.04 (64.38, 517.22) | 0.484 |
| DCA | 143.22 (6.85, 338.59) | 122.26 (17.87, 245.46) | 0.723 |
| NCA | 3.95 (2.65, 6.80) | 3.51 (2.44, 5.17) | 0.431 |
| DHCA | 1.50 (0.48, 2.79) | 1.54 (0.61, 3.32) | 0.710 |
| 7,12-diketoLCA | 0.27 (0.11, 0.41) | 0.23 (0.08, 0.44) | 0.966 |
| 7-KDCA | 2.73 (1.19, 7.86) | 1.93 (1.07, 3.23) | 0.122 |
| 12-keto-CDCA | 11.53 (4.87, 18.46) | 6.26 (3.44, 14.59) | 0.312 |
| 3-DHCA | 0.23 (0.07, 1.88) | 0.46 (0.0001, 1.18) | 0.210 |
| UCA | 8.33 (4.14, 11.85) | 6.89 (5.39, 8.95) | 0.762 |
| 3β-CA | 5.40 (3.96, 13.99) | 4.22 (2.82, 7.08) | 0.229 |
| α-MCA/ω-MCA | 0.99 (0.24, 1.95) | 0.74 (0.20, 1.43) | 0.250 |
| β-MCA | 0.42 (0.0002, 1.23) | 0.33 (0.0002, 1.29) | 0.598 |
| HCA | 10.51 (4.42, 25.51) | 9.15 (4.65, 17.36) | 0.494 |
| CA | 59.23 (25.44, 303.33) | 52.08 (26.69, 138.15) | 0.484 |
| GLCA | 1.42 (0.0002, 6.40) | 0.71 (0.0001, 2.74) | 0.106 |
| GUDCA | 122.24 (70.66, 340.18) | 143.73 (78.61, 347.48) | 0.856 |
| GHDCA | 9.92 (5.40, 21.31) | 8.11 (6.46, 13.38) | 0.505 |
| GCDCA | 478.14 (305.15, 1060.06) | 439.05 (247.20, 807.78) | 0.401 |
| GDCA | 77.33 (15.54, 163.90) | 64.95 (8.67, 128.21) | 0.672 |
| LCA-3S | 1.40 (0.0002, 7.08) | 0.50 (0.0001, 2.31) | 0.154 |
| GDHCA | 0.79 (0.70, 1.05) | 0.80 (0.63, 1.006) | 0.829 |
| GHCA | 6.62 (3.73, 10.14) | 5.84 (3.67, 9.26) | 0.697 |
| GCA | 130.94 (69.46, 329.06) | 127.34 (60.83, 215.93) | 0.373 |
| UDCA-3S | 3.01 (0.82, 7.06) | 2.59 (1.66, 4.92) | 0.517 |
| CDCA-3S | 1.79 (0.43, 6.28) | 1.79 (0.70, 3.81) | 0.842 |
| DCA-3S | 0.14 (0.0002, 4.70) | 0.37 (0.0001, 1.32) | 0.562 |
| TLCA | 2.10 (1.21, 3.84) | 1.92 (1.45, 2.81) | 0.634 |
| TUDCA | 2.88 (1.33, 10.02) | 3.97 (1.57, 7.91) | 0.842 |
| THDCA | 0.08 (0.0002, 1.29) | 0.0002 (0.0001, 0.22) | 0.058 |
| TCDCA | 48.31 (27.59, 101.37) | 54.85 (21.63, 90.82) | 0.672 |
| TDCA | 6.09 (0.45, 21.96) | 6.93 (0.61, 12.93) | 0.856 |
| GLCA-3S | 115.52 (9.60, 267.63) | 37.19 (7.66, 154.57) | 0.337 |
| T-α-MCA/T-β-MCA | 1.83 (0.37, 4.78) | 1.09 (1.15, 4.80) | 0.672 |
| T-β-MCA | 1.61 (0.73, 2.76) | 1.25 (0.90, 2.78) | 0.979 |
| THCA | 0.76 (0.33, 1.41) | 0.90 (0.54, 1.30) | 0.789 |
| TCA | 11.33 (3.92, 33.25) | 8.04 (4.19, 19.09) | 0.401 |
| GUDCA-3S | 50.44 (26.98, 95.77) | 61.59 (34.65, 90.52) | 0.462 |
| GCDCA-3S | 57.14 (44.89, 141.23) | 69.22 (33.26, 103.32) | 0.789 |
| GDCA-3S | 38.86 (6.85, 82.55) | 48.16 (4.27, 101.53) | 0.789 |
| GCA-3S | 0.004 (0.0001, 1.12) | 0.10 (0.0002, 0.50) | 0.897 |
| TLCA-3S | 6.44 (0.60, 26.48) | 4.17 (1.56, 15.37) | 0.421 |
| CDCA-3Gln | 20.82 (7.93, 102.09) | 17.80 (8.74, 50.59) | 0.897 |
| TUDCA-3S | 2.47 (0.95, 4.54) | 3.29 (2.17, 4.30) | 0.243 |
| TCDCA-3S | 9.54 (4.59, 20.02) | 9.65 (4.24, 17.07) | 0.938 |
| TDCA-3S | 2.27 (0.51, 6.39) | 4.29 (0.23, 9.69) | 0.829 |
| GCDCA-3Gln | 47.36 (25.88, 123.72) | 40.90 (29.24, 78.71) | 0.484 |
| GDCA-3Gln | 18.45 (8.73, 61.74) | 18.54 (8.52, 42.77) | 0.562 |

The Mann-Whitney U test was used for comparisons between the two groups.
